# Bioengineered stem cell membrane functionalized nanoparticles combine anti-inflammatory and antimicrobial properties for sepsis treatment

**DOI:** 10.1186/s12951-023-01913-3

**Published:** 2023-05-26

**Authors:** Lu Lu, Lingli Quan, Jian Li, Junbin Yuan, Xinmin Nie, Xueyuan Huang, Hang Dong, Yanrong Su, Yufen Huang, Qingjie Kou, Leping Liu, Haiting Liu, Xionghui Zhou, Rong Gui, Lan Gu

**Affiliations:** 1 Department of Blood Transfusion, The Third Xiangya Hospital, Central South University, Changsha, 410013 Hunan China; 2 Department of Urology, The Xiangya Hospital, Central South University, Changsha, 410008 Hunan China; 3 Department of Laboratory Medicine, The Third Xiangya Hospital, Central South University, Changsha, 410013 Hunan China; 4 Department of Pediatrics, The Third Xiangya Hospital, Central South University, Changsha, 410013 Hunan China; 5 Department of Pulmonary and Critical Care Medicine, The Affiliated Zhuzhou Hospital of Xiangya Medical College, Central South University, Zhuzhou, 412007 China

**Keywords:** sepsis, Cytokine storm, Antibacterial, Metal-organic framework, Mesenchymal stem cells

## Abstract

**Background:**

Sepsis is a syndrome of physiological, pathological and biochemical abnormalities caused by infection. Although the mortality rate is lower than before, many survivors have persistent infection, which means sepsis calls for new treatment. After infection, inflammatory mediators were largely released into the blood, leading to multiple organ dysfunction. Therefore, anti-infection and anti-inflammation are critical issues in sepsis management.

**Results:**

Here, we successfully constructed a novel nanometer drug loading system for sepsis management, FZ/MER-AgMOF@Bm. The nanoparticles were modified with LPS-treated bone marrow mesenchymal stem cell (BMSC) membrane, and silver metal organic framework (AgMOF) was used as the nanocore for loading FPS-ZM1 and meropenem which was delivery to the infectious microenvironments (IMEs) to exert dual anti-inflammatory and antibacterial effects. FZ/MER-AgMOF@Bm effectively alleviated excessive inflammatory response and eliminated bacteria. FZ/MER-AgMOF@Bm also played an anti-inflammatory role by promoting the polarization of macrophages to M2. When sepsis induced by cecal ligation and puncture (CLP) challenged mice was treated, FZ/MER-AgMOF@Bm could not only reduce the levels of pro-inflammatory factors and lung injury, but also help to improve hypothermia caused by septic shock and prolong survival time.

**Conclusions:**

Together, the nanoparticles played a role in combined anti-inflammatory and antimicrobial properties, alleviating cytokine storm and protecting vital organ functions, could be a potential new strategy for sepsis management.

**Supplementary Information:**

The online version contains supplementary material available at 10.1186/s12951-023-01913-3.

## Background

Sepsis is defined as a life-threatening organ dysfunction caused by dysregulated host response to infections [1]. The host immune response in sepsis involves complex pathophysiology. Infection may trigger a cytokine-mediated excessive inflammatory response, associating with tissue damage, endothelial cell dysfunction and organ failure [2]. Currently, in the absence of targeted therapies in clinic, sepsis has traditionally been treated by implementing the supportive therapies. Clinical guidelines propose that antibiotic therapy is the standard care for sepsis. However, clinical data suggests immunosuppression and persistent infection in more than 60% of survivors after antibiotic therapy, greatly affecting patients’ outcomes [3]. So, it’s urge for us to develop new therapeutic strategies for sepsis.

Infection and inflammation both play an important role in the pathogenesis of sepsis. When pathogens invade, the host immune system is activated to eradicate them. For example, pattern recognition receptors (PRRs) on host immune cells recognize microbial pathogen-associated molecular patterns (PAMPs), and then release pro-inflammatory cytokines (such as TNF-*α*, IL-1β, and IL-6), activate vascular endothelial cells, increase the expression of adhesion molecules, and recruit leukocytes to eliminate foreign invasion [4, 5]. Therefore, the combined delivery of antibiotic and anti-inflammatory agents to the lesions induced by invading bacteria may be a novel strategy for sepsis treatment that can simultaneously reduce bacterial transmission and avoid excessive inflammation [4]. Simultaneous delivery of multiple drugs requires a suitable carrier that efficiently loads drugs and targets bacteria-induced tissue lesions as well as achieving effective release. Metal-organic framework (MOFs) are novel hybrid porous materials synthesized from metal ions or metal clusters and organic ligands [6]. In this study, we used the silver metal-organic framework (AgMOF), which formed by coordination binding of 2-methylimidazole and silver nitrate, as a nano drug carrier. The AgMOF has superior properties, such as well-defined pore aperture, tailorable composition and structure, tunable size, versatile functionality and high agent loading, making it promising as a candidate for drug delivery [7]. Besides, Silver ions are known to have satisfactory anti-inflammatory and antimicrobial effects, while also enhancing the therapeutic efficacy of antibiotic [8].

Sepsis can be caused by bacteria, viruses, fungi, etc., in which bacterial sepsis is the most common. In bacterial sepsis, recognition of PAMPs by specific cell surface receptors is critical for activating the immune system and precipitating inflammatory responses [9], including Toll-like receptors (TLRs), nucleotide-binding oligomerization domain like receptors (NLRs), C-type lectinreceptor (CLRs), etc. Specifically, the Receptor for Advanced Glycation End Products (RAGE) is a major cell surface molecule involved in advanced glycation end products (AGEs) toxicity and plays a crucial role in inflammatory responses. RAGE is expressed in many cell types involving the innate immune system and is able to recognize various endogenous molecules released in various inflammatory and injurious conditions [10]. As a key molecule in HMGB1/RAGE axis, RAGE mediates caspase-11-dependent pyroptosis and death in endotoxemia and sepsis, thereby affecting disease progression [11]. Previous studies have shown that inhibiting RAGE can have a strong protective effect in mice subjected to bacterial sepsis [12, 13], suggesting that RAGE is a potential new target for sepsis treatment. Here, FPS-ZM1, a novel high-affinity RAGE-specific small molecule inhibitor, was simultaneously loaded in AgMOF to effectively inhibit RAGA binding to the ligands, thus reducing inflammatory responses.

BMSCs have been widely used in preclinical and clinical trials for a variety of diseases due to their unique immunomodulatory, anti-inflammatory, anti-apoptotic and anti-microbial properties, having become suitable candidates for regenerative medicine and tissue engineering [14, 15]. It’s has demonstrated that BMSCs can improve many pathophysiological processes critical for sepsis, such as immune imbalance and coagulation disorders, reducing the incidence of organ failure and death [16, 17]. However, many challenges need to be overcome to successfully apply stem cell therapy to sepsis, including neoplastic transformation, immune rejection, and pathogen transmission [18]. Recently, the extracellular vesicle-sized cell membrane coating nanotechnology has been an emerging platform to address many shortcomings of cell therapy [15]. In this study, we used the intact natural cell membrane from BMSCs for nanoparticle functionalization in order to avoid the potential risk caused by direct application of BMSCs and mimic the natural properties of the source cells. On the one hand, molecules retained on the BMSCs membrane can play a key role in immune regulation by mediating cell-cell contact mechanisms, including cell adhesion molecule 1 (ICAM-1), vascular cell adhesion protein 1 (VCAM-1), and inhibitory molecule programmed death 1 (PD-1)[19]. On the other hand, the expression and function of adhesion molecules, chemokine receptors and matrix metalloproteinases (MMPs) on the membrane surface are essential for the migration of BMSCs to damaged or diseased tissues [20].

Lipopolysaccharide (LPS), the major component of the outer membrane of Gram-negative bacteria, is one of the most potent immunostimulatory compounds known in nature, being a key constituent in sepsis by overactivating the innate immune system [21]. It has been shown that BMSCs treated with LPS can enhance the nutritional effects and functional properties to protect against harsh inflammatory environments [22]. For example, Pardis et al. modified BMSCs with LPS (LPS-BMSCs), which increased anti-apoptotic and anti-inflammatory activities of BMSCs, as well as their bacterial clearance capacity in septic mice [23]. We hypothesized that BMSCs could exert immune-cell-like effects, that is, “short-term memory” to danger signals and environmental stimuli, possibly related to altered expression of key molecules on their membrane surface (such as adhesion molecules, chemokine receptors)[24].

**Fig. 1:**
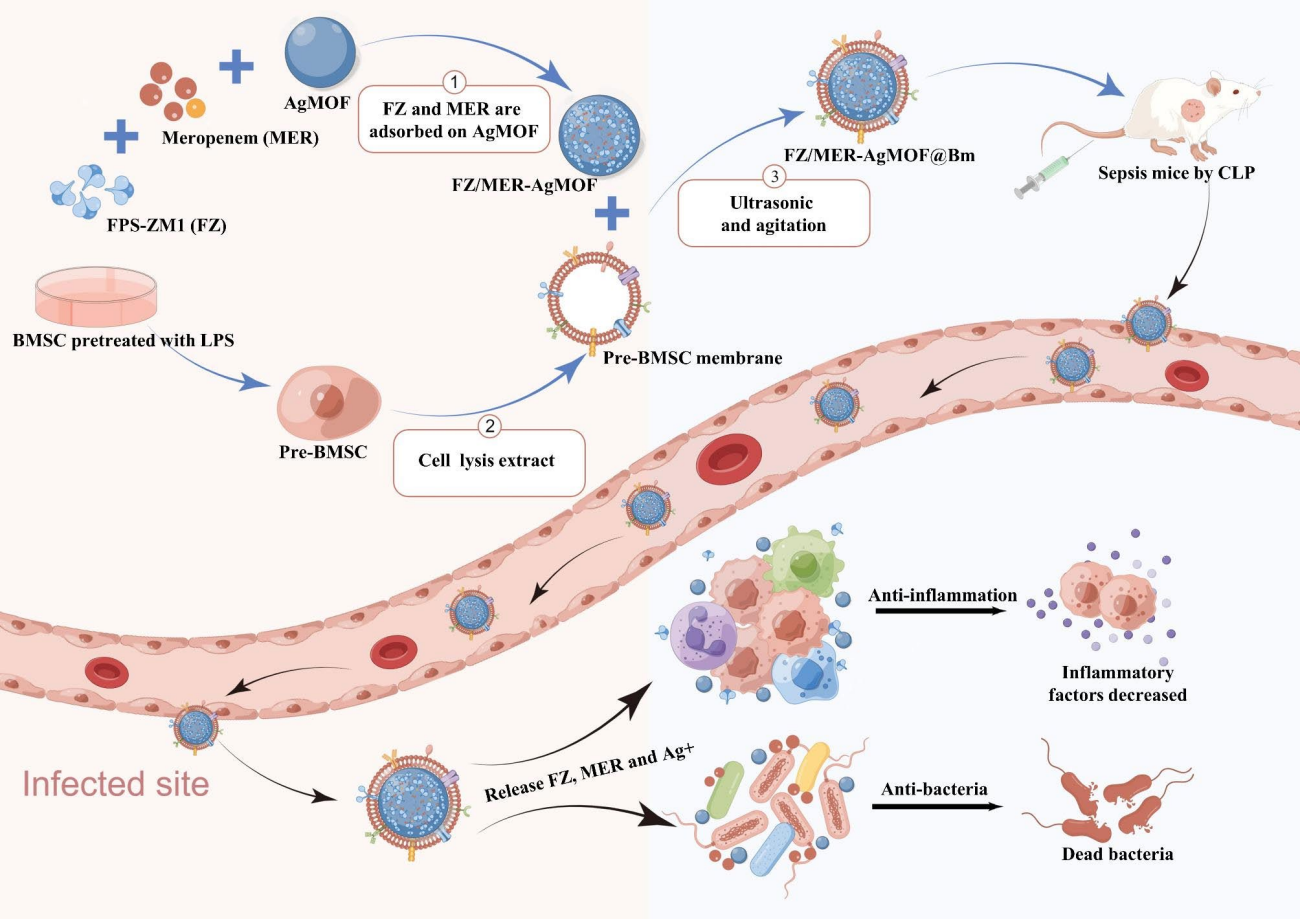
Schematic diagram of FZ/MER-AgMOF@Bm in the treatment for sepsis

Taken together, our study intends to design and construct a novel nano-drug loading system for the sepsis therapy, FZ/MER-AgMOF@Bm. The nanoparticles were modified with LPS-BMSCs membranes, using Ag-MOF as the nanocore, while loading FPS-ZM1 and the antibiotic meropenem to exert dual anti-inflammatory and antibacterial effects and improve the efficacy of sepsis (Fig. 1).

## Results

### Preparation and characterization of FZ/MER-AgMOF@Bm

The preparation of FZ/MER-AgMOF@Bm was divided into three steps: (1) extraction of membrane vesicles from preconditioning BMSCs (BMSCm); (2) preparation of FZ/MER-AgMOF; (3) construction of functionalized BMSC-like metal organic framework, FZ/MER-AgMO@Bm. Specifically, AgMOF was synthesized using 2-methylimidazole as organic ligand and silver nitrate as metal source, and it was used as a drug carrier to load the small molecule inhibitor FPS-ZM1 (FZ) and the antibiotic meropenem (MER) using magnetic stirring and electrostatic adsorption to obtain FZ/MER-AgMOF. Then, membrane vesicles derived from BMSCs were fused with FZ/MER-AgMOF by ultrasonic extrusion to finally achieve the construction of FZ/MER-AgMOF@Bm.

#### Characterization of LPS-BMSCs

**Fig. 2:**
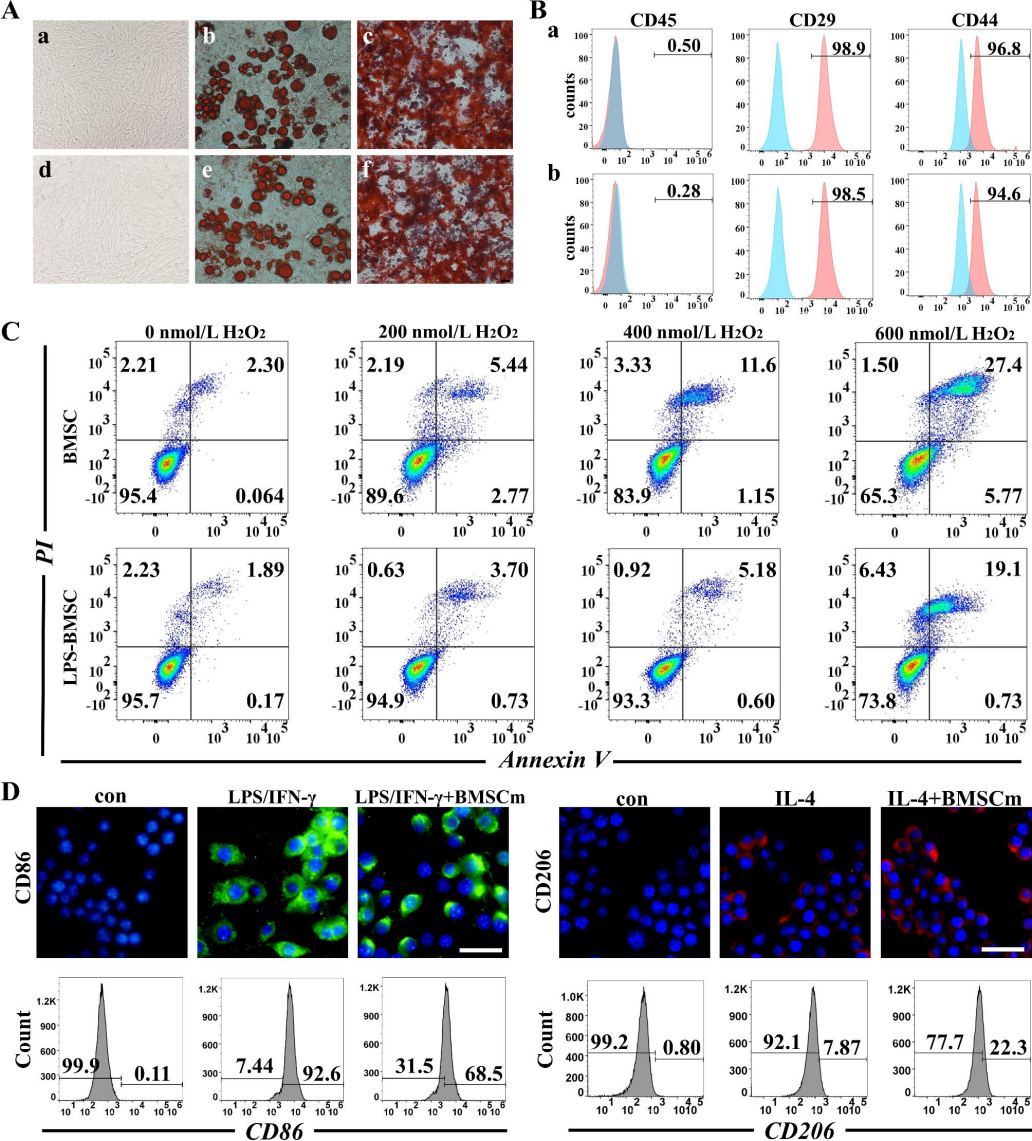
Characterization of LPS-BMSCs. **(A)** The morphology, adipogenic and osteogenic differentiation of BMSCs(a-c) and LPS-BMSCs(d-f). Scale bar: 50 μm. **(B)** Flow cytometric analysis of the immunophenotype of BMSCs(a) and LPS-BMSCs(b). Blue filled lines indicate lgG controls, red filled lines the respective antibodies. **(C)** Apoptosis of BMSCs and LPS-BMSCs treated with H_2_O_2_ for 24 h assessed by Flow cytometry. **(D)** RAW264.7 stimulated with LPS (100 ng/mL) +IFN-*γ* (20 ng/mL) or IL4 (20 ng/mL) treated with or without LPS-BMSC membrane vesicles (BMSCm) for 24 h. Blue: DAPI; Green:CD86 (M1 marker); Red:CD206 (M2 marker). Scale bar: 50 μm.

Here, we primed BMSCs with 1 µg/mL LPS for 24 h [23]. The characteristics of LPS-BMSCs were verified by morphological monitoring, differentiation potential, and immunophenotyping. After LPS primed, the morphology of BMSCs did not change, and their differentiation potential during osteogenesis and adipogenesis was also normal (Fig. 2A). In addition, flow cytometry showed that LPS-BMSCs were positive for BMSC markers (CD44 and CD29) and negative for hematopoietic markers (CD45) (Fig. 2B). Briefly, these results indicate that the BMSCs pretreated with LPS maintain the original cell characteristics and meet the BMSCs identification criteria. LPS can activate BMSCs and enhance their cell viability and anti-apoptotic ability to protect against external adverse stimuli [22]. To investigate the anti-apoptosis effect of LPS-BMSCs, BMSCs and LPS-BMSCs were exposed to H_2_O_2_. Flow cytometry showed that the apoptosis rate of LPS-BMSCs was significantly lower than that of BMSCs under various concentrations of H_2_O_2_, indicating that LPS priming could increase the anti-apoptosis ability of BMSCs (*P* < 0.05, Fig. 2C and S1). Besides, previous studies have shown that BMSCs can promote macrophages polarization to M2 through direct cell-cell contact or paracrine [25]. Next, we determined whether BMSCm can alter M2-like polarization of macrophages. Polarization corroboration performed by immunofluorescence and flow cytometric analysis to examine the levels of polarization-related surface markers. The results showed that BMSCm decreased the expression of M1 marker (CD86 and CD16/32) and increased the level of M2 marker (CD206) in macrophages (Fig. 2D, S2 and S3), which suggested that BMSCm still has some ability to promote macrophages polarize to M2.

#### Characterization of FZ/MER-AgMOF@Bm

**Fig. 3:**
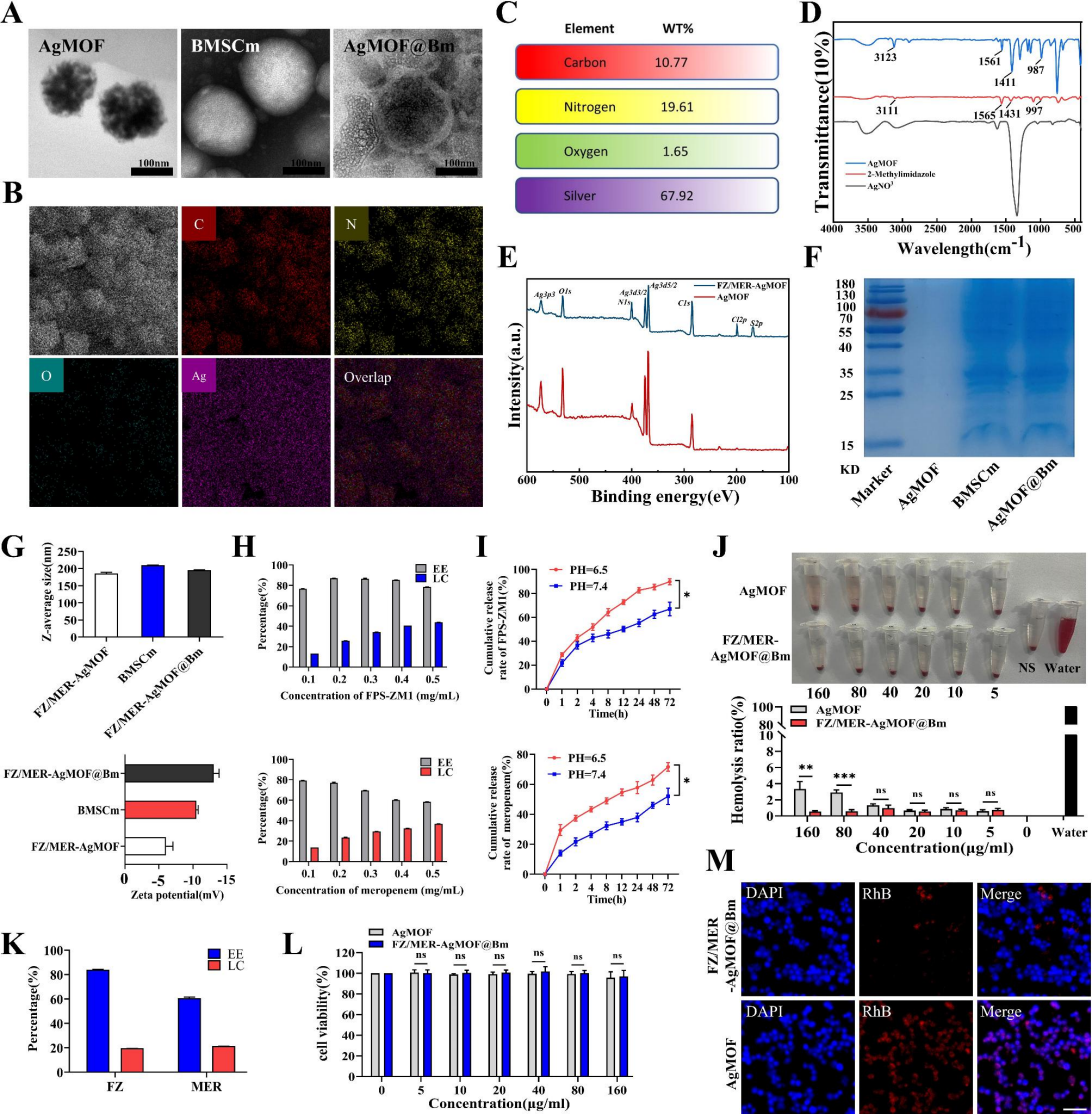
Characterizations of FZ/MER-AgMOF@Bm. **(A)** TEM images of AgMOF, LPS-BMSC membrane vesicles (BMSCm) and AgMOF@Bm. **(B)** SEM elemental mapping images of AgMOF. **(C)** Relative quantification of elements in Figure B. **(D)** FTIR spectra of AgMOF, 2-methylimidazole and AgNO_3_. **(E)** XPS survey spectra of FZ/MER-AgMOF and AgMOF. **(F)** SDS‑PAGE protein assessment for AgMOF, BMSCm and AgMOF@Bm. **(G)** DLS analysis of hydrodynamic diameter and zeta potential for FZ/MER-AgMOF@Bm, BMSCm and FZ/MER-AgMOF. **(H)** EE and LE of FPS-ZM1 and meropenem in AgMOF. **(I)** Cumulative release rate of FPS-ZM1 and meropenem from FZ/MER-AgMOF@Bm at pH 6.5 or pH 7.4. **(J)** Hemolysis ratios of erythrocyte’ suspensions treated with different concentrations of AgMOF or FZ/MER-AgMOF@Bm for 2 h. **(K)** EE and LE of FZ/MER-AgMOF@Bm. **(L)** The cell viability (%) of RAW264.7 treated with Ag-MOF or FZ/MER-AgMOF@Bm for 24 h. **(M)** Images of RAW264.7 incubated with FZ/MER-AgMOF@Bm or AgMOF for 12 h. Scale bar: 50 μm. Data are derived from three independent experiments and presented as mean ± SEM in the bar graphs. Values of controls were normalized to 1. *P < 0.05, **P < 0.01, ***P < 0.001, ns: not significant

Next, different characterization techniques were used to detect the properties of functionalized nanoparticles and the key intermediates. Transmission electron microscope (TEM) shows that AgMOF was a loose pore structure with irregular spherical edges, BMSCm was spherical, while FZ/MER-AgMOF@Bm presented a unique shell core structure, which directly indicates that BMSCm was successfully encapsulated on the nano core (Fig. 3A). Energy dispersive spectroscopy (EDS) analysis shows that AgMOF contains key elements C, N, O and Ag, with relative contents of 10.77%, 19.61%, 1.65% and 67.92% respectively (Fig. 3B C). Furthermore, fourier transform infrared spectroscopy (FTIR) demonstrated that AgMOF was successfully synthesized, with a characteristic peak at 3123 cm^-1^ caused by C-H stretching vibration on the imidazolium positive ring, while the peak at 1561 cm^-1^ represented the imidazolium ring backbone vibration (C = C or C = N); in addition, the peak near 1411 cm ^-1^ may be the deformation vibration peak of C-H on methyl (Fig. 3D). Next, the X-ray photoelectron spectroscopy (XPS) profile showed that compared with AgMOF, FZ/MER AgMOF has more elements Cl and S (Fig. 3E), which derived from FPS-ZM1 and meropenem, indicating that FZ/MER-AgMOF was successfully prepared. The sodium dodecyl sulfate polyfate acrylamide gel electrophoresis (SDS-PAGE) result showed that almost all BMSC membrane proteins were preserved in AgMOF@Bm, further revealing successful encapsulation of LPS-BMSCs-derived membrane coatings (Fig. 3F). Finally, dynamic light scattering (DLS) analysis showed that the mean hydrated particle size of FZ/MER-AgMOF@Bm was 194.93 ± 1.11 nm, which was slightly larger than that of FZ/MER-AgMOF (185.33 ± 2.81 nm). The mean zeta potential of FZ/MER-AgMOF@Bm was − 13.07 ± 0.62 mV, which was similar to BMSCm (-10.5 ± 0.22 mV), but significantly lower than that of FZ/MER-AgMOF (-6.04 ± 0.82 mV), reflecting the successful encapsulation of BMSCm on nanoparticles (Fig. 3G).

For optimizing the drug loading of the nanoparticles, the drug/metal organic framework ratio was adjusted. As shown in Fig. 3H, loading capacity (LC) increased from 13.29 to 43.96% when FPS-ZM1 increased from 0.1 mg/mL to 0.5 mg/mL (equivalent to 20% and 100% (w/w) of the drug/polymer ratio), and entrapment efficiency (EE) also exhibit a parabolic trend. For meropenem, LC increased from 13.70 to 36.90% and EE decreased from 79.37 to 58.48%. Overall, LC and EE of FPS-ZM1 were slightly higher than meropenem, possibly due to the more hydrophobic nature of FPS-ZM1. To load both drugs simultaneously, concentrations of FPS-ZM1 and meropenem were fixed at 0.2 mg/mL and 0.3 mg/mL, respectively. High performance liquid chromatography (HPLC) analysis showed that FPS-ZM1’s LC and EE in FZ/MER-AgMOF@Bm were 19.74 ± 0.02% and 84.03 ± 0.13%, while meropenem’s were 21.42 ± 0.19% and 60.71 ± 0.69% (Fig. 3K). An ideal drug carrier should be able to load drug effectively and reach specific sites for response release. Compared with healthy tissues and extracellular environment, infection site and intracellular environment are slightly acidic environment [26]. If the acidic environment can promote the drug release from nanoparticles, the drug concentration at specific sites can be effectively increased. To test this end, a neutral blood circulation environment and an acidic infection microenvironment were simulated with release medium at pH 7.4 and pH 6.5, respectively. Notably, FPS-ZM1 and meropenem were more easily released from FZ/MER-AgMOF@Bm in pH 6.5 (Fig. 3I), which was beneficial to increase the drug concentration at the infection site.

#### Biocompatibility and safety of FZ/MER-AgMOF@Bm in vitro

For evaluating the biocompatibility of FZ/MER-AgMOF@Bm in vitro, hemolysis rate was tested. Incubated with 5% red blood cell (RBC) for 2 h, FZ/MER-AgMOF@Bm did not cause obvious hemolysis, and its resulting hemolysis rate (< 2%) was lower than the standard of International Organization for Standardization (ISO, 5%). And at high concentrations (≥ 80 µg/mL), the hemolysis rate was significantly lower caused by FZ/MER-AgMOF@Bm than by AgMOF (*P* < 0.05, Fig. 3J), indicating the good biocompatibility of nanoparticles. It has been reported that the mechanism of silver related nanoparticles induced hemolysis may be related to factors such as the release of silver ions and the direct interaction between particles themselves and RBCs [27], which may also be the possible cause of AgMOF induced hemolysis. In addition, FZ/MER-AgMOF@Bm did not affect the viability of the RAW264.7 (Fig. 3L), demonstrating its’ low cytotoxicity. Recent studies have established that biomimetic nanoparticles have good immune escape ability and reduce drug phagocytosis by macrophages during circulation [28]. We assessed the immune evasion ability of FZ/MER-AgMOF@Bm by examining macrophage phagocytosis of nanoparticles. As shown in Fig. 3M, a clear red fluorescence was monitored in RAW264.7 cells treated with AgMOF. In contrast, red fluorescence was significantly reduced in macrophages in FZ/MER-AgMOF@Bm group, suggesting that nanoparticles have reduced immunogenicity after encapsulation with cell membrane vesicles, which is beneficial to reduce phagocyte recognition and clearance.

### In vitro effect of FZ/MER-AgMOF@Bm

#### Anti-inflammatory effect of FZ/MER-AgMOF@Bm

**Fig. 4:**
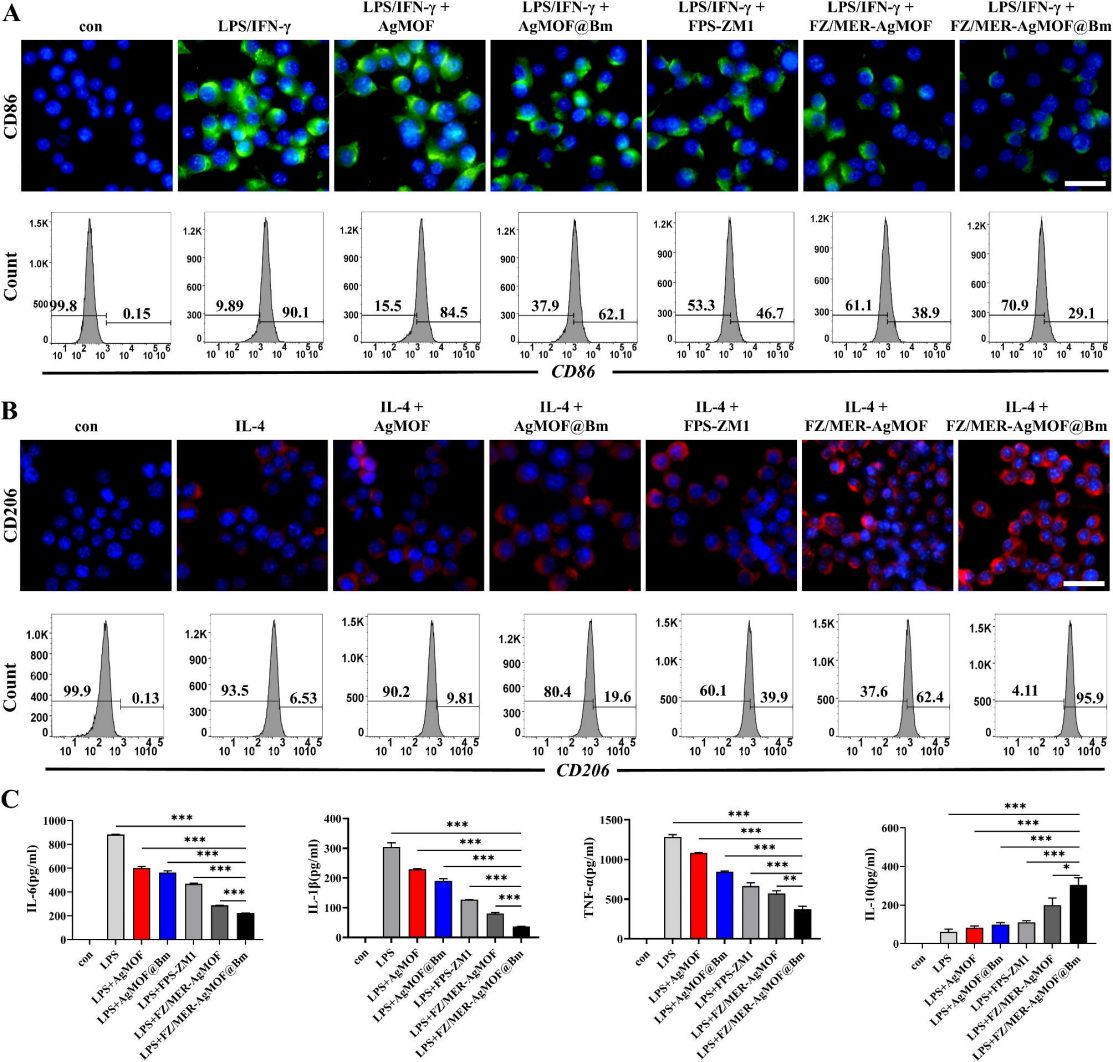
The anti-inflammatory effect of FZ/MER-AgMOF@Bm in vitro. **(A)** RAW264.7 were stimulated with LPS (100 ng/mL) + IFN-γ (20 ng/mL) for M1-like polarization and treated indicated reagents for 24 h. **(B)** RAW264.7 were stimulated with IL-4 (20 ng/mL) for M2-like polarization and treated with indicated reagents for 24 h. Blue: DAPI; Green:CD86 (M1marker); Red:CD206 (M2 marker). Scale bar: 50 μm. **(C)** ELISA for IL-6, IL-1β, TNF-α and IL-10 in the supernatants of indicated reagents-primed RAW 264.7 stimulated with LPS (1 µg/mL) for 24 h. Data are derived from three independent experiments and presented as mean ± SEM in the bar graphs. *P < 0.05, **P < 0.01, ***P < 0.001

A recent study establishes that BMSCs have an immunomodulatory effect and influence the phenotype and functions of macrophages [25]. To determine whether the nanoparticles can alter M1-like or M2-like polarization of macrophages, we used immunofluorescence and flow cytometry to detect the macrophage markers, including CD16/32 [29, 30], CD86 and CD206 [31]. Compared with other groups, FZ/MER-AgMOF@Bm exposure most significantly reduced levels of CD86 and CD16/32 in M1-like macrophages (Fig. 4A, S4 and S5), whereas the expression of CD206 was up-regulated (Fig. 4B and S4), suggesting that the nanoparticles can promote macrophage polarization to M2 while inhibiting M1 polarization. In addition, we investigated the role of FZ/MER-AgMOF@Bm in the transformation of macrophages from M1 to M2. To this end, we incubated macrophages with LPS (100 ng/mL) plus IFN-γ (20 ng/mL) for 24 h to polarize the cells to an M1-like phenotype, and then added nanoparticles for additional 24 h. Polarization corroboration by flow cytometric analysis was performed to examine the level of polarization-related surface marker, and the result showed that compared with LPS + IFN-*γ* group (M1 positive control group), treatment with FZ/MER-AgMOF@Bm resulted in a dramatic increase in the expression level of CD206 (*P* < 0.05, Fig. S6). These findings established FZ/MER-AgMOF@Bm could mildly repolarize M1 macrophages to the M2 phenotype, with FPS-ZM1 playing a key synergistic role. As we known, the M1 are pro-inflammatory while the M2 are anti-inflammatory. ELISA showed that the pro-inflammatory factors (IL-1β, IL-6 and TNF-α) were significantly decreased while the inflammatory suppressors (IL-10) were increased in the FZ/MER-AgMOF@Bm group (*P* < 0.05, Fig. 4C). In line with above, this finding also suggests that FPS-ZM1 and nanocomplexes can effectively promote anti-inflammatory polarization in macrophages.

#### Antibacterial effect of FZ/MER-AgMOF@Bm

**Fig. 5:**
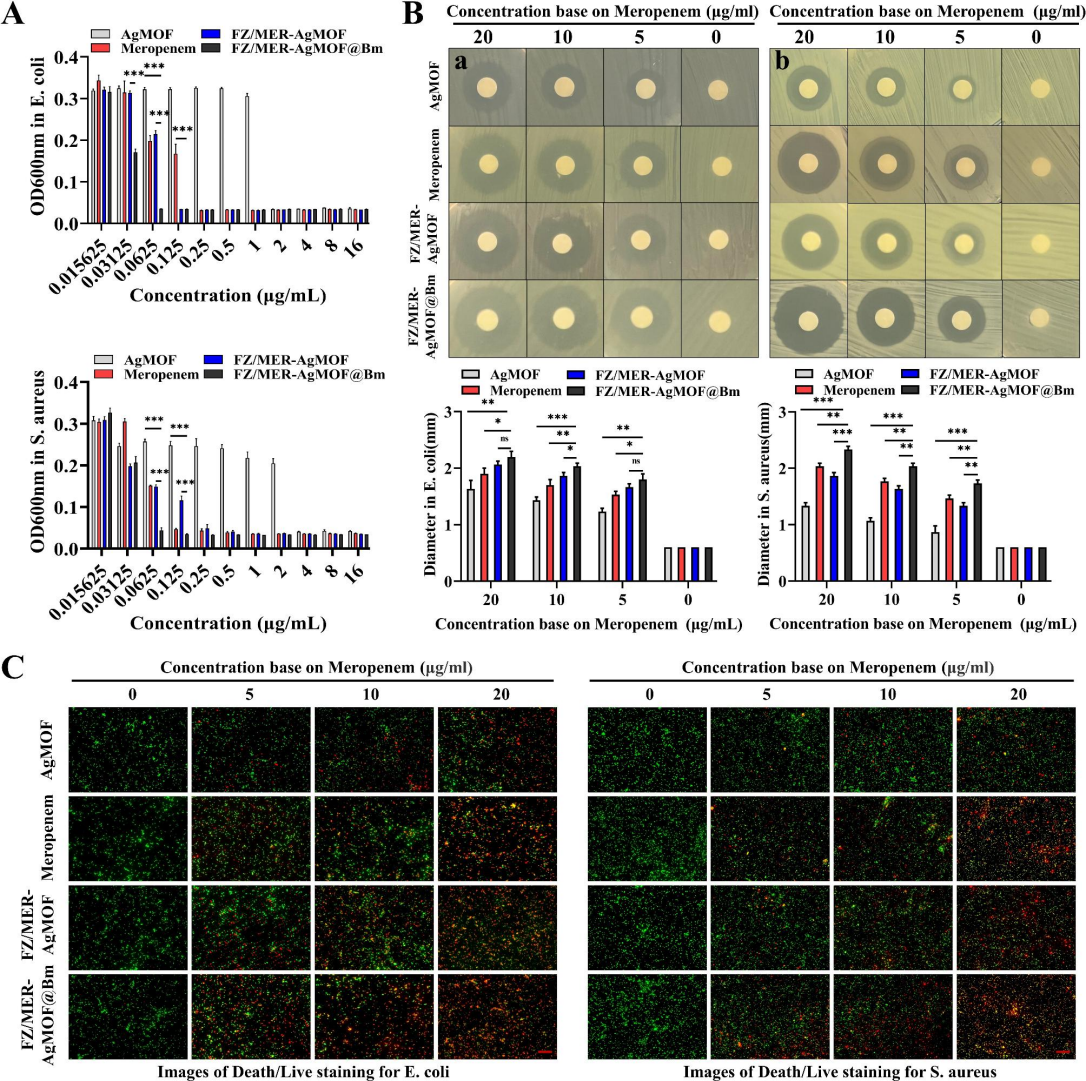
The effect of FZ/MER-AgMOFB@m against bacteria in vitro. **(A)** MIC values of reagents at different concentrations against E. coli and S. aureus for 18 h. **(B)** Inhibition zones and corresponding inhibition zone diameters of different reagents against E. coli (a) and S. aureus (b) for 18 h. **(C)** Images of Death/Live staining after exposing E. coli and S. aureus to varying concentrations of different reagents for 18 h. Green: living bacteria; Red: dead bacteria. Scale bar: 40 μm. Data are derived from three independent experiments and presented as mean ± SEM in the bar graphs. *P < 0.05, **P < 0.01, ***P < 0.001, ns: not significant

In addition to the above anti-inflammatory effects, FZ/MER-AgMOF@Bm also exerts antibacterial effects, due to its own AgMOF and loaded meropenem. The minimum inhibitory concentration (MIC) is a measure of the antimicrobial activity of an antimicrobial agent, which refers to the lowest drug concentration that it takes to inhibit growth of bacteria in vitro [32]. Considering that gram-negative bacteria are the main pathogens that induce sepsis, which are usually related to the pathogenesis of severe sepsis and septic shock [33], we used E. coli for bacteriostasis test. First, the MICs of AgMOF, MER, FZ/MER-AgMOF and FZ/MER-AgMOF@Bm were 2, 0.25, 0.125 and 0.0625 µg/mL, respectively (Fig. 5A). While disk diffusion method showed that FZ/MER-AgMOF@Bm had a good inhibitory effect on the growth of E. coli (Fig. 5B). In addition, fluorescence staining of live/dead bacteria showed that the number of dead bacteria increased dose-dependently after FZ/MER-AgMOF@Bm treatment (Fig. 5C). In fact, sepsis can also be caused by gram-positive bacteria in clinical, so we further explored the antibacterial effect of the nanoparticles on S. aureus. For S. aureus, the MICs of AgMOF, MER, FZ/MER-AgMOF and FZ/MER-AgMOF@Bm were 4, 0.125, 0.25 and 0.0625 µg/mL, respectively (Fig. 5A). Disk diffusion method and live/dead bacteria staining showed that FZ/MER-AgMOF@Bm had satisfactory inhibitory and bactericidal effects on the growth of S. aureus (Fig. 5B and C).

### Biodistribution of FZ/MER-AgMOF@Bm in vivo

**Fig. 6:**
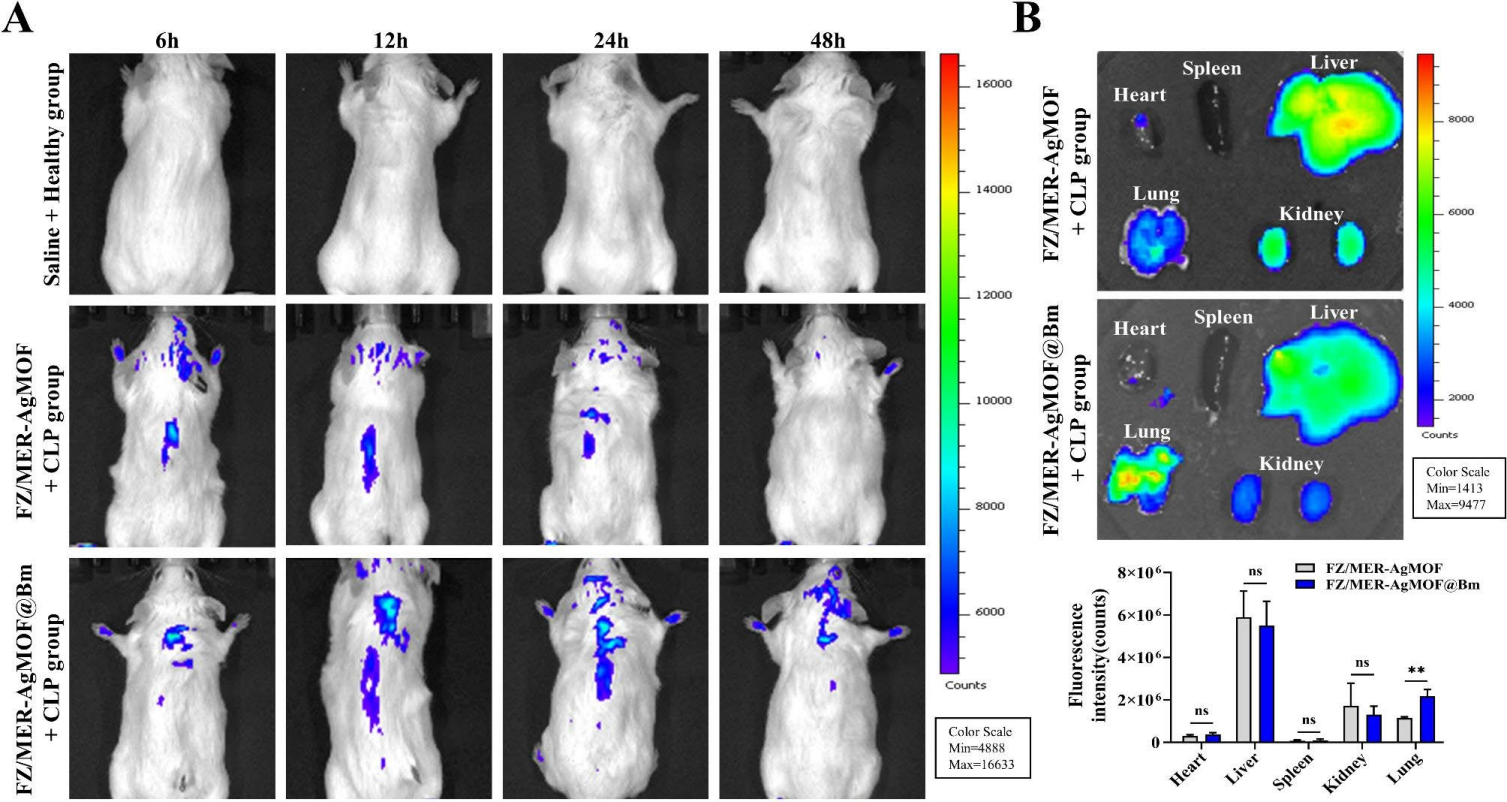
The distribution of FZ/MER-AgMOF@Bm in vivo. **(A)** In vivo fluorescence images in CLP mice at 6, 12, 24 and 48 h upon intravenous treatment with Cy5-labeled FZ/MER-AgMOF or Cy5-labeled FZ/MER-AgMOF@Bm. **(B)** Ex vivo bioluminescent images and semiquantitative assessment of fluorescence signals of main organs at 24 h after treatment with Cy5-labeled FZ/MER-AgMOF or Cy5-labeled FZ/MER-AgMOF@Bm. Data are derived from three independent experiments and presented as mean ± SEM in the bar graphs. *P < 0.05. ns: not significant

The biological distribution and biocompatibility of nanoparticles in animals are crucial for subsequent therapeutic effect research and clinical translation. To investigate the distribution of nanoparticles in vivo, Cy5-labeled FZ/MER-AgMOF and Cy5-labeled FZ/MER-AgMOF@Bm were injected into CLP mice via the tail vein. IVIS spectrum-chromatography indicated that both FZ/MER-AgMOF and FZ/MER-AgMOF@Bm gradually accumulated in livers within 24 h after injection, but were basically excreted from CLP mice after 48 h. In addition, there was a significant difference in the distribution of FZ/MER-AgMOF and FZ/MER-AgMOF@Bm in the lungs, with the latter accumulating more in the lung (Fig. 6A). In line with this, in vitro organ imaging analysis also showed that the accumulation of FZ/MER-AgMOF@Bm was significantly more than FZ/MER AgMOF in lung (*P* < 0.05) (Fig. 6B). These findings suggesting that FZ/MER-AgMOF@Bm may tend to accumulate in the lung and contribute to the recovery of lung injury in sepsis. Besides, FZ/MER-AgMOF@Bm accumulated largely in the liver, but rarely in the kidney, indicating that the nanoparticles may be mainly metabolized in the liver while a small amount in the kidney.

### Safety evaluation of FZ/MER-AgMOF@Bm in vivo

**Fig. 7:**
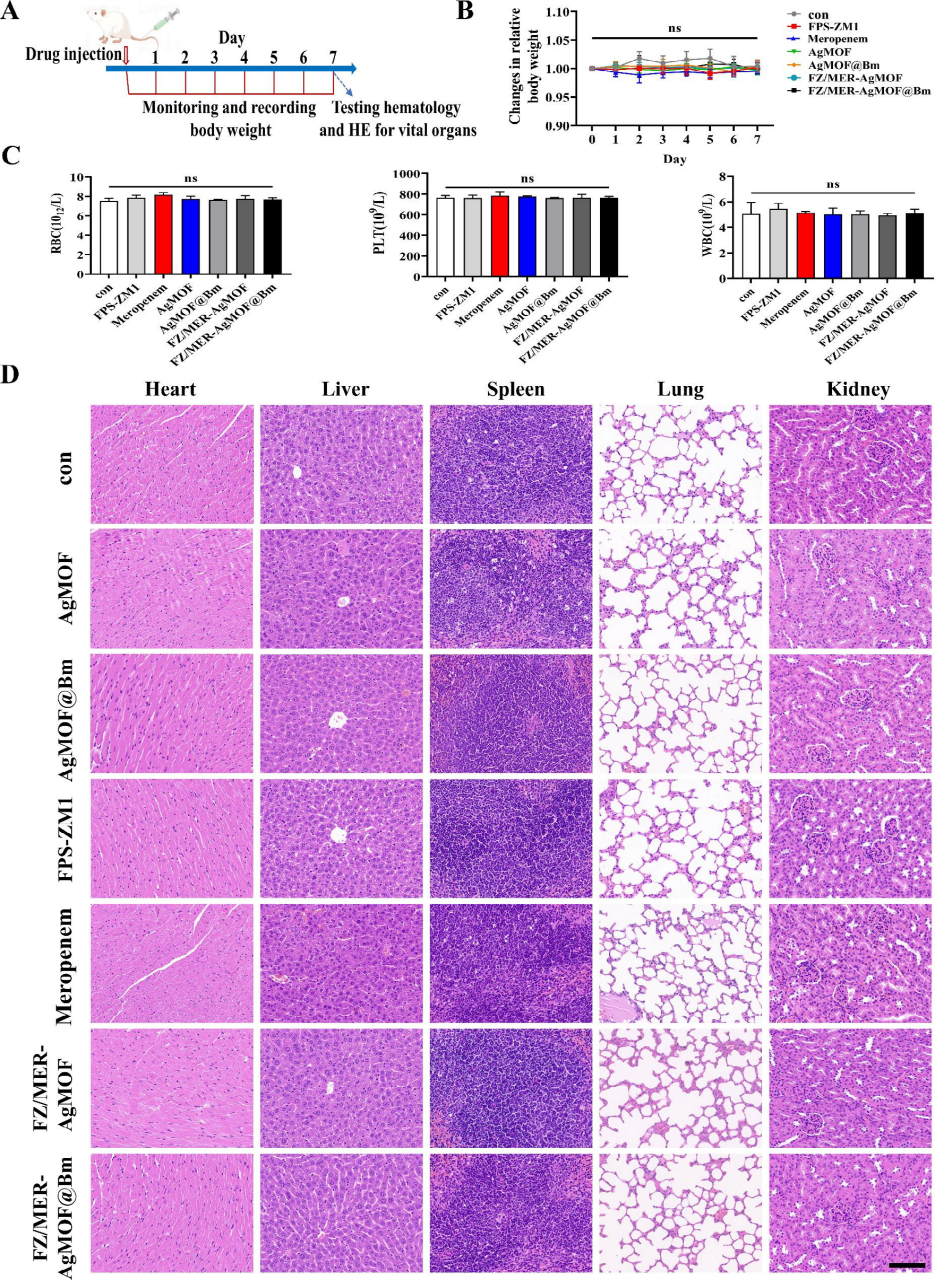
The biocompatibility evaluation of FZ/MER-AgMOF@Bm in vivo. **(A)** Schematic diagram of in vivo toxicity experimental design (n = 5). **(B)** Mice weight changes after injecting with normal saline, AgMOF (10 mg/kg), AgMOF@Bm (10 mg/kg), FPS-ZM1 (2 mg/kg), meropenem (2 mg/kg), FZ/MER-AgMOF (FPS-ZM1 dose of 2 mg/kg) or FZ/MER-AgMOF@Bm (FPS-ZM1 dose of 2 mg/kg) during 1 week. **(C)** Representative images of H&E staining for vital organs from mice at 1 week after intravenous injection of different reagents. Scale bar: 100 μm. **(D)** Complete blood count (CBC) for mice at 1 week after intravenous injection of different reagents. Data are derived from three independent experiments and presented as mean ± SEM in the bar graphs. ns: not significant

To test the potential toxicity of FZ/MER-AgMOF@Bm in vivo, survival experiments were conducted. Different agents (AgMOF, AgMOF@Bm, FPS-ZM1, meropenem, FZ/MER-AgMOF, FZ/MER-AgMOF@Bm) were injected into mice via tail vein, and related parameters were observed (Fig. 7A). The results showed that the nanoparticles did not affect the weight of mice during 7 days after injection (Fig. 7B). Besides, complete blood count (CBC) at one week after intravenous injection was normal, indicating that FZ/MER-AgMOF@Bm had no significant effect on RBC, white blood cells (WBC), and platelets (PLT) (Fig. 7C). Furthermore, in order to further evaluate the biosafety of FZ/MER-AgMOF@Bm in vivo, we studied the effects of nanoparticles and free drugs on important organs. The results showed that inflammation, necrosis, fibrosis and histological abnormalities were not observed in any organ in each group (Fig. 7D). The above results showed that FZ/MER-AgMOF@Bm had low toxicity and good biocompatibility in vivo, and had the potential to be a safe and low toxic anti-infective nanomedicine.

### Protection of mice from septic lethality by FZ/MER-AgMOF@Bm in vivo

**Fig. 8:**
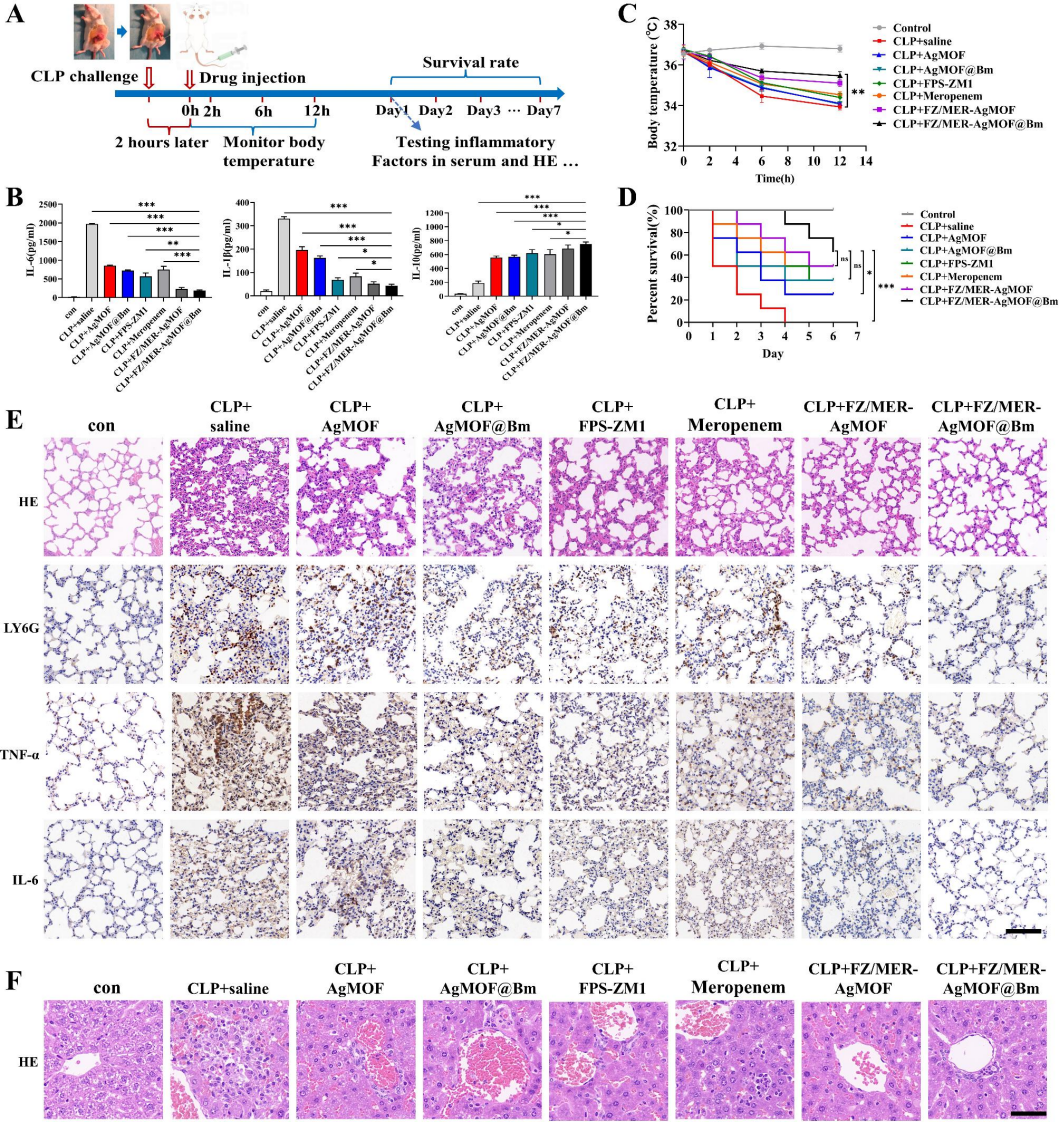
Therapeutic efficacy of FZ/MER-AgMOF@Bm against CLP-induced sepsis in mice. **(A)** Schematic diagram of experimental design for sepsis therapy. **(B)** Plasma IL-1β, IL-6 and IL-10 levels from mice injected with normal saline, AgMOF (7.5 mg/kg), FPS-ZM1 (1.5 mg/kg), meropenem (1.5 mg/kg), FZ/MER-AgMOF (FPS-ZM1 dose of 1.5 mg/kg) or FZ/MER-AgMOF@Bm (FPS-ZM1 dose of 1.5 mg/kg) 2 h after CLP challenge (n = 5). **(C)** Body temperature change curves of mice receiving different treatments (n = 5). **(D)** Kaplan-Meier survival plots for each treatment group (n = 4 of control group, n = 8 of other treatment groups). **(E)** Representative images of H&E staining and IHC staining of LY6G, TNF-α and IL-6 in lungs (n = 5). **(F)** Representative images of H&E staining for livers (n = 5). con: Sham mice treating with saline; CLP: cecal ligation and puncture; Scale bar: 100 μm. Data are derived from three independent experiments and presented as mean ± SEM in the bar graphs. *P < 0.05, ***P < 0.001, ns: not significant

The therapeutic effect of FZ/MER-AgMOF@Bm was investigated in a cecal puncture ligation (CLP) sepsis model. Here, mice were treated with different drugs, including saline, AgMOF, AgMOF@Bm, FPS-ZM1, meropenem, FZ/MER-AgMOF, and FZ/MER-AgMOF@Bm, 2 h after CLP challenge (Fig. 8A). Because cytokine storm induced multiple organ failure is a major cause of severe sepsis in mice [34]. The levels of inflammatory factors in serum were measured in CLP mice to determine the effect of nanoparticles on inflammatory responses in sepsis. Consistent with these findings in vitro, FZ/MER-AgMOF@Bm significantly decreased the pro-inflammatory factors (IL-1β and IL-6) and increased the inflammatory inhibitory factor (IL-10) in CLP mice, and this effect was significantly better than free drugs and FZ/MER-AgMOF (Fig. 8B). Changes in body temperature in mice are an important feature of sepsis ,which reflect the severity of the disease and impairment of immune responses after infection [35]. In this study, we monitored the body temperature of mice within 12 h after treatment with different drugs, and result showed that CLP challenge led to a rapid decline of body temperature (36.6 ℃ to 33.9 ℃) in control mice while FZ/MER-AgMOF@Bm exhibited a strong protection effect on body temperature decrease in mice (from 36.7 ℃ to 35.5 ℃), indicating that the nanoparticles had the effect of improving hypothermia in sepsis (Fig. 8C). In terms of survival time, CLP mice died within 4 days after saline injection, while FZ/MER-AgMOF@Bm significantly prolonged the survival time of CLP mice, and their 6-day survival rate mentioned about 60%, indicating the protective effect of this nanoparticle in a model of sepsis (Fig. 8D).

Organ injury caused by inflammatory reaction is the main cause of sepsis deterioration and death. Among them, the lung is the first and the most common organ of failure during sepsis [36]. As mentioned above, FZ/MER-AgMOF@Bm accumulated in lungs. To determine whether the nanoparticles have a protective effect on lung injury, we performed H&E staining and immunohistochemistry on lung tissue. We observed that FZ/MER-AgMOF@Bm significantly reduced the thickness of alveolar wall in the lungs of CLP mice. Moreover, immunohistochemical images and their quantitative analysis results showed that the infiltration of neutrophils (LY6G) and the secretion of proinflammatory factors (TNF-α and IL-6) in lung tissue was reduced after FZ/MER-AgMOF@Bm injection, suggesting that infection-related inflammation was effectively relieved (Fig. 8E and S7). As an important metabolic and detoxifying organ, liver injury will affect the pharmacokinetics of nanoparticles in vivo. Here, liver injury in septic mice was further assessed. H&E staining showed that hepatic perivascular inflammatory cell recruitment was attenuated in FZ/MER-AgMOF@Bm group (Fig. 8F), suggesting that the nanoparticles can also alleviated liver injury.

**Fig. 9:**
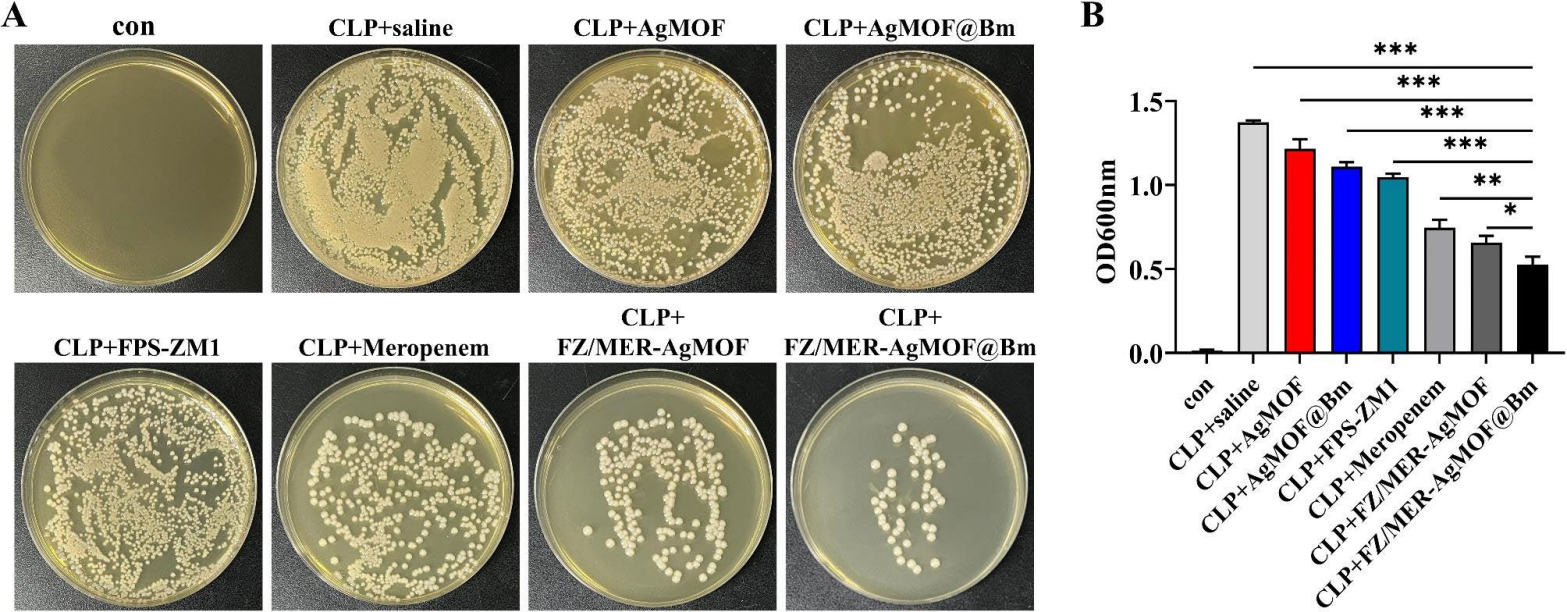
In vivo antibacterial effects of FZ/MER-AgMOF@Bm. **(A)** Representative images of bacterial colonization in the blood of mice received different treatment (n = 5). **(B)** Bacterial count in peritoneal lavage fluid of mice received different treatment (n = 5). con: Sham mice treating with saline; CLP: cecal ligation and puncture. Data are derived from three independent experiments and presented as mean ± SEM in the bar graphs. *P < 0.05, **P < 0.01, ***P < 0.001

Furthermore, the in vivo anti-bacterial effect of FZ/MER-AgMOF@Bm was studied. Whole blood was collected from mice in different treatment groups, and serum was obtained after standing and spread in luria-bertani (LB) culture dishes to observe the number of bacteria in the blood of mice. It was uncovered that compared to the other groups, the bacterial counts in FZ/MER-AgMOF@Bm group were the most significantly reduced, evidencing accelerated bacterial clearance in CLP mice (Fig. 9A). In addition, peritoneal lavage fluid was obtained from different treatment groups, and OD_600_ values of the lavage fluid were measured using a UV spectrophotometer to estimate bacterial density. As shown in Fig. 9B, peritoneal lavage fluid from mice treated with FZ/MER-AgMOF@Bm contained the least number of bacteria.

## Discussion

Sepsis is systemic inflammation due to excessive activation of the innate immune system caused by microbial infection, and subsequent organ failure are the main causes of death. Although the pathogenesis of sepsis is intricate, infection and excessive activation of host innate immunity are key factors in its pathogenesis [4]. After pathogen invasion, host immune system is activated, and immune cells (such as macrophages) can recognize lipopolysaccharide on gram-negative bacilli through Toll like receptors (TLRs), and release proinflammatory factors (such as TNF-α, IL-1β and IL-6), thus causing damage of the microvascular endothelium, continuous hypotension and organ failure [37]. At present, the treatment of sepsis mainly depends on antibiotics. However, the acute physiological changes during sepsis could lead to poor pharmacokinetics and unsuccessful drug delivery [38]. The poor effect of drug treatment has led to the abuse of antibiotics, which has virtually increased the toxicity, drug resistance and double infection of patients. Therefore, it is necessary to invent new therapeutic methods.

Anti-infective drugs or anti-inflammatory drugs alone can only address some of the causes of sepsis and may even limit the therapeutic effect [5]. In this study, we constructed a nano-delivery system, FZ/MER-AgMOF@Bm, that can be used to deliver drugs in combination. Space-time co-existence of bacterial and inflammatory responses is used as a therapeutic breakthrough to locally sequester bacterial infections, thereby avoiding systemic spread of bacteria and excessive activation of the immune system. First, Ag NPs, called as “nano antibiotic”, acts as antimicrobial agents against a large number of gram-negative and positive bacteria, as well as resistant pathogens [39]. In this study AgMOF-based antimicrobial drug delivery was also shown to enhance the therapeutic efficacy of antibiotics, helping to reduce the dosage of antibiotics and the side effects caused by the use of antibiotics. Second, FPS-ZM1 loaded with FZ/MER-AgMOF@Bm is a high-affinity but non-toxic RAGE-specific inhibitor to be shown to inhibit inflammatory responses in a variety of diseases, including kidney injury, Alzheimer’s disease, neuroinflammation, and tumors [40]. Shen C et al. demonstrated that FPS-ZM1 significantly inhibited RAGE-dependent microglial activation, nuclear translocation of nuclear factor-kappaB p65 (p65 NF-κB), and expression of downstream inflammatory mediators such as TNF-α and IL-1β, which in turn reduced oxidative stress and inflammatory response to play a neuroprotective role [41]. Besides, FPS-ZM1 can also significantly inhibit ischemia-induced inflammatory response and reduce endothelial injury by blocking the HMGB1/RAGE axis [42]. Therefore, FPS-ZM1 is used in the treatment of septic mice to suppress excessive inflammatory responses, providing new insights into anti-inflammatory treatment of sepsis.

Modern studies demonstrated that macrophage with M2-phenotype exhibited prominent anti-inflammatory effect through secreting IL-10, arginase 1(Arg-1) and transforming growth factor beta (TGF-β), playing important roles in alleviating inflammation and repairing damaged tissues [43]. Recently, increasing evidence has demonstrated that increased M1/M2 polarized macrophage ratio is closely related to sepsis, and a decrease in the M1/M2 ratio is beneficial for improving organ dysfunction in sepsis [44]. Our study has testified that FZ/MER-AgMOF@Bm can promote M2 polarization of macrophages, which may be related to BMSCm and FPS-ZM1. It has shown that BMSCs express immunosuppressive ligands (such as programmed cell death protein-1 and Fas-ligands) and TLRs on their membranes in response to inflammatory stimuli. These ligands can bind to receptors on the surface of immune cells and then affect the function of cells, including macrophages [45]. Furthermore, it was suggested that RAGE activation could mediate M1-phenotype polarization via HMGB1-RAGE-MAPK axis, and inhibition of RAGE contributed to decrease M1/M2 polarization ratio and alleviate inflammation [46]. It has been suggested that FPS-ZM1 can prevent M1-like macrophage polarization by effectively inhibiting the RAGE signaling pathway, while promoting anti-inflammatory polarization (M2), which in turn reduces pro-inflammatory factor levels (IL-1β, IL-6 and TNF-α), while the expression level of IL-10, which is an anti-inflammatory suppressor, is increased [47, 48]. IL-10 is thought to be an important mediator of the compensatory anti-inflammatory response that occurs in response to intense inflammation [49]. In this study, the level of IL-10 in the CLP group was significantly higher than that in the control group, which may be related to the compensatory anti-inflammatory response of the mice in the inflammatory state. Moreover, both FPS-ZM1 and nanocomposites could promote M2-like polarization in vitro, with FZ/MER-AgMOF@Bm showing the strongest effect, which may be the underlying reason why the nanoparticles increased the expression level of IL-10. In animal models of severe sepsis, elevated levels of IL-10 expression are crucial for limiting tissue damage and improving host survival [50].

Lung is the most vulnerable organ during the development of sepsis, and patients with sepsis often present with acute lung injury (ALI) or acute respiratory distress syndrome (ARDS)[44, 51]. Despite advances in management strategies, the prognosis of ALI in patients with sepsis is still poor. In our study, compared with nanoparticles without BMSCm camouflage modification, FZ/MER-AgMOF@Bm accumulated better in lungs and alleviated sepsis-induced lung injury and prolonged the survival time of mice, which may be related to the function of multiple molecules on the surface of BMSCs. It has been shown that BMSCs express multiple chemokine receptors and integrins on their surface, allowing them to target sites of inflammation and injury [52]. In addition, in lung injury models, BMSCs have a high homing rate to inflammatory and injury sites in the lung, which is related to the function of a variety of molecules expressed on their cell membranes [53]. For example, multiple chemokine receptors (CXCR1, CXCR2, CXCR3, CXCR4 and CXCR5 etc.) expressed on the surface of BMSCs can interact with signaling factors released from injured tissues and then enable BMSCs to home to the inflammation and injury sites [45]. Furthermore, adhesion molecules on BMSC membranes under inflammatory stimuli can directly interact with damaged endothelial cells in the lung and participate in the maintenance of endothelial barrier integrity by preserving endothelial barrier proteins [54]. In addition, the lung represents an organ where RAGE is abundantly expressed [13], thus the delivery of FPS-ZM1 to the lung to inhibit RAGE may be an effective measure to attenuate lung injury in sepsis.

## Conclusions

In summary, FZ/MER-AgMOF@Bm is a potential novel nano-delivery strategy for the treatment of sepsis, which not only shows good therapeutic effects in reducing pro-inflammatory cytokine levels and protecting organ damage, but also helps to improve hypothermia caused by septic shock and improve survival. Moreover, the BMSCs membrane camouflage strategy proposed in this study is suitable for in vivo application of a variety of nanoparticles and may aid in the treatment of various diseases. In addition, this work provides a strategy for combined delivery of multiple drugs that promises to provide new ideas for the treatment and prevention of various infectious diseases, especially in the epidemic of infectious diseases.

## Materials and methods

### Materials

Silver nitrate (AgNO_3_) and 2-methylimidazole were purchased from Aladdin (China). FPS-ZM1 and ultrapure LPS were purchased from Med Chem Express (USA). Meropenem was purchased from TOPSCIENCE (China). PE/Cy7-conjugated anti-CD44, PE-conjugated anti-CD45, FITC-conjugated anti-CD29, PE-conjugated anti-CD86, Alexa Fluor 647-conjugated anti-CD206 and FITC-conjugated anti-CD16/32 antibodies were purchased from Biolegend (USA). Zombie Aqua™ Fixable Viability Kit was purchased from Biolegend (USA). Transcription Factor Buffer Set was purchased from BD Biosciences (USA). Annexin V-FITC/PI apoptosis detection and mesenchymal stem cells adipogenic or osteogenic differentiation and staining kit was produced by Dalian Meilun Biotech Co., Ltd. (China). The mouse IL-1β, TNF-α, IL-6 and IL-10 ELISA kit were purchased from Thermofisher (USA). The rat anti-CD86 and anti-CD16/32 antibody was purchased from Proteintech (China), and rabbit anti-CD206 antibody was provided by Cell Signaling Technology (USA). The LIVE/DEAD backlight bacterial viability kit, YF647 -linked goat anti-rabbit IgG and YF488-conjugated goat anti-mouse IgG were purchased from US Everbright Inc. (China). Anti-TNF-α, anti-IL-6, anti-LY6G antibodies and hematoxylin and eosin (H&E) were purchased from Servicebio Technology (China). Fetal bovine serum (FBS), Dulbecco’s modified Eagle medium (DMEM) (high glucose), DMEM/F-12, and trypsin were purchased from Life Technologies (USA). Cy5, rhodamine B (RhB), and DAPI were provided by Yeasen Biotechnology (China).

### Cells and animal models

RAW264.7 cells were bought from the Advanced Research Center, Central South University. BMSCs were purchased from Dalian Meilun Biotech Co., Ltd. (China), which were obtained from bone marrow of Balb/c mice. Cells were cultured in DMEM (or DMEM/F-12) containing 1% streptomycin/penicillin together with 10% FBS in a 37 °C humidified 5% CO_2_ incubator.

For animal studies, ICR mice (female, 6~8 weeks old) were bought from Hunan SJA Laboratory Animal Co., Ltd. (China), and housed with ad libitum food/water under specific pathogen-free conditions. All animal experiments were approved by the Experimental Animal Ethics Committee of Central South University and performed according to the requirements of National Act on the Use of Experimental Animals (People’s Republic of China). A model of polymicrobial sepsis induced by cecal ligation and puncture (CLP) was established by the method reported in the literature [55].

### Characterization of LPS-BMSCs

First, BMSCs were treated with 1 *µ*g/mL LPS for 24 h, and the morphology of cells was observed by microscopy. Then, the immunophenotypes of treated and untreated BMSCs were determined by flow cytometry evaluating CD44, CD45 and CD29. Besides, the multipotency of BMSCs was confirmed by osteogenic and adipogenic differentiation. Alizarin red staining and Oli Red O staining were used to detect the differentiation of LPS-BMSCs and BMSCs during osteogenesis and adipogenesis. In addition, BMSCs and LPS-BMSCs were treated with H_2_O_2_ at various concentrations (0, 200, 400 and 600 nmol/mL) for 24 h. Then cell viability was evaluated using an Apoptosis Kit following the manufacturer’s instructions. Briefly, the cells were dyed with Annexin V and PI and analyzed using flow cytometry.

### Synthesis of FZ/MER-AgMOF@Bm

First, AgNO_3_ (10 mg) and 2-methylimidazole (0.194 g) were added to 5 mL of doble distilled water (ddH_2_O), respectively. AgNO_3_ solution was added dropwise to a 2-methylimidazole solution and allowed to react for 5 min at room temperature with magnetic stirring until milky white. The above solution was centrifuged at 10,000 rpm for 5 min, washed three times with ddH_2_O and freeze-dried in a vacuum freeze-dryer to form AgMOF.

Next, 0.5 mg AgMOFs was dissolved in 1 mL ddH_2_O, meropenem and FPS-ZM1 were added at a certain mass ratio, magnetic stirring was performed overnight at room temperature, and FZ/MEM-AgMOF was obtained by centrifugation.

For membrane vesicle preparation, LPS-BMSCs were resuspended in ddH_2_O cracked by 0.25 mM ethylene diamine tetraacetic acid (EDTA) with protease inhibitors at 4 ℃ for 1 h. The cells were then lysed sufficiently by repeated freeze-thaw procedures. After the cell disruption solution was centrifuged at 2,000 rpm, 4 ℃ for 10 min, the supernatant was taken and further centrifuged at 20,000 rpm for 30 min to obtain LPS-BMSCs membrane debris. Finally, the solution containing LPS-BMSCs membrane debris was passed through porous polycarbonate membrane with different pore sizes (1 μm、800 nm、400 nm and 200 nm) and repeatedly squeezed for 10 cycles to obtain LPS-BMSC membrane vesicles (BMSCm).

Finally, BMSCm were ultrasonically fused (5 min, 42 kHz, 100 W) with an equal amount of FZ/MEM-Ag-MOF. This mixed solution was filtered 20 times through a porous polycarbonate membrane with a pore size of 200 nm and then excess BMSCm were removed by centrifugation to obtain FZ/MEM-AgMOF@Bm.

### Characterization of FZ/MER-AgMOF@Bm

Morphology characterization of AgMOF, BMSCm and FZ/MER-AgMOF@Bm was captured through a transmission electron microscope (TEM) with a Tecnai G2 Spirit TEM (FEI, USA). Elemental mapping analysis was performed by Scanning Electron Microscope (SED, Sigma 300, Germany) with an integrated Super-X EDS system. The Zetasizer Nano ZS (Malvern Nano series, Malvern, UK) was used to measure zeta potential and hydrodynamic diameter. The elemental composition of FZ/MER-AgMOF was assessed by X-ray photoelectron spectroscopy (XPS, ESCALAB250Xi, USA). The fourier transform infrared spectroscopy (FTIR) was performed to study the molecular functional groups of AgMOF. The BMSC membrane proteins were identified by sodium dodecyl sulfate-polyacrylamide gel electrophoresis (SDS-PAGE).

The drug loading content (LC) and encapsulation efficiency (EE) were measured by high performance liquid chromatography (HPLC, Agilent 1260, USA). For parameter settings, the chromatographic column was Agilent Eclipse XDB-C18 (250 × 4.6 mm), and the detection wavelength was set at 254 nm. The mobile phase consisted of a mixture of water and ACN (0 min: 90/10, v/v; 8 min: 10/90, v/v; 17 min: 10/90, v/v; 17.1:90/10, v/v; 20 min: 90/10, v/v) at 35 °C with a flow rate of 1.0 mL/min. Besides, the calculation formulas for LE and EE were as follows: EE = (quality of drugs contained on nano-carrier/total amount of drugs used) × 100%; LE = (mass of drug contained on nano-carrier/mass of nano-carrier) ×100%.

### Drug release in vitro

The release profiles of FPS-ZM1 and meropenem in vitro were analyzed using the dialysis method at 37 °C. Specifically, 2 mg of FZ/MER-AgMOF@Bm was dispersed in 2 mL of ddH_2_O into a dialysis bag (cutoff molecular weight 3.5 kDa), which was immersed in 12 mL of release medium (PBS buffer) at pH 6.5 or pH 7.4. Then, sample was placed in a shaker (100 rpm, 37 °C). At different time points (1, 2, 4, 8, 12, 24, 48, 72 h), 0.2 mL of release medium was taken from the container for HPLC analysis, and equatorial release medium was added into the container to keep the volume unchanged.

### Cytotoxicity and biocompatibility in vitro

For cytotoxicity assay, RAW264.7 cells were seeded in 96-well plates at a density of 2 × 10^3^ cells per well for overnight culturing, and then the different concentrations (0, 5, 10, 20, 40, 80 and 160 µg/mL) of NPs (AgMOF and FZ/MER-AgMOF@Bm) were added for 24 h incubation. Then, 10 µL of CCK-8 reagent was added, followed by a 3 h incubation. Finally, the absorbance at 450 nm was measured to calculate the cell viability.

For biocompatibility assay, we evaluated hemolysis rate and macrophage phagocytosis for AgMOF and FZ/MER-AgMOF@Bm. Firstly, 5% red blood cell suspension was prepared from fresh mouse blood. Then the different concentrations (0, 5, 10, 20, 40, 80 and 160 µg/mL) of NPs were incubated with 5% red blood cell suspension for 2 h at 37 ℃. Following centrifugation at 1,500 rpm for 10 min, supernatants were collected and absorbance was measured at 545 nm while ultrapure water and PBS were used as positive and negative controls. To detect the immune escape ability of NPs, RAW264.7 cells were seeded in a 6-well plate and incubated with rhodamine B-labeled FZ/MER-AgMOF@Bm or AgMOF for 12 h, and then the phagocytic fluorescence of macrophages was observed by using a fluorescence microscope.

### Cytokine secretion and macrophage polarization analyses

RAW264.7 cells were seeded in 6-well plates for overnight culturing, and were treated with PBS and different drugs (AgMOF, AgMOF@Bm, FPS-ZEM, meropenem, FZ/MER-AgMOF and FZ/MER-AgMOF@Bm) for 2 h, and then stimulated with 1 µg/mL LPS for additional 24 h. Subsequently, cytokine levels (IL-1β, TNF-α, IL-6 and IL-10) were measured by using ELISA kits. To test the inhibition of FZ/MER-AgMOF@Bm on M1 macrophage polarization, RAW264.7 cells were stimulated with LPS (100 ng/mL) plus IFN-γ (20 ng/mL) for 24 h to display the M1 phenotype. At the same time, the cells were treated with PBS, AgMOF, AgMOF@Bm, FPS-ZM1, FZ/MER-AgMOF and FZ/MER-AgMOF@Bm in the experimental groups. In addition, RAW264.7 cells were stimulated with 20 ng/mL IL-4 for 24 h for M2 macrophage differentiation. During polarization, macrophages were further treated with different drugs to assess FZ/MER-AgMOF@Bm effects on M2 polarization. The polarization transitions were evaluated with immunofluorescence and flow cytometry analysis.

### In vitro antimicrobial assay

All bacteria were purchased from Haibo Biotechnology Co., Ltd (China): Staphylococcus aureus (S. aureus, ATCC29213), Escherichia coli (E. coli, ATCC25922). For disk-diffusion assays, a single colony of each bacterium was dispersed in normal saline and OD_600_ value of bacteria was adjusted to 0.8 ~ 1. Then, bacterial solution was evenly spread on Mueller-Hinton (MH) medium, and the filter discs with 6 mm diameter were placed on the surface of MH agar plate. Different concentrations of AgMOF, meropenem, FZ/MER-AgMOF, and FZ/MER-AgMOF@Bm in a volume of 10 µL were dropped into the filter discs. Plates were incubated for 18 h at 37 ℃ in an incubator.

MIC values of antimicrobial agents against different bacteria were tested as follow method. We added a high concentration of the drug to the bacterial solution (OD_600_ value of 0.8 ~ 1), and the final total volume of the bacterial solution was 2 mL. Then, 1 mL of the above bacterial solution was added to the bacterial solution without drug, with a total volume of 2 mL. According to this method, obtain bacterial solution containing different drug concentrations (0.015625, 0.03125, 0.0625, 0.125, 0.25, 0.5, 1, 2, 4, 8 and 16 µg/mL). Then, 200 µL bacteria solution was added to wells in 96-well plates. The bacteria solution without antimicrobial agents was selected as the control group. These plates were incubated for 18 h at 37 °C. The OD_600_ values were measured to detect the bacterial growth and the lowest drug concentration that completely inhibited bacterial growth in the wells was taken as MIC.

For LIVE/DEAD bacterial viability assays, the two strains were inoculated via a similar procedure used for MIC studies. After incubation for 18 h at 37 °C, the treated bacteria were centrifuged to obtain the sediment, and then were stained with a LIVE/DEAD backlight bacterial viability kit. The images were photographed by a fluorescence microscope.

### In vivo biocompatibility evaluation

In this study, the biocompatibility NPs was verified in terms of body weight, complete blood bount, and H&E staining of major organs. First, mice were randomly divided into control group (saline injection), AgMOF, AgMOF@Bm, FPS-ZM1, meropenem, FZ/MER-AgMOF and FZ/MER-AgMOF@Bm, with 5 mice in each group. Then, 100 µL saline containing AgMOF (10 mg/kg), AgMOF@Bm (10 mg/kg) FPS-ZM1 (2 mg/kg), meropenem (2 mg/kg), FZ/MER-AgMOF (FPS-ZM1 dose of 2 mg/kg) or FZ/MER-AgMOF@Bm (FPS-ZM1 dose of 2 mg/kg) were injected into mice via the tail vein. Mice were continuously monitored for body weight for 1 week. Then, the mice were sacrificed and their blood was taken to detect blood routine. Mouse tissue was fixed in PBS containing 4% paraformaldehyde and sectioned after embedding in paraffin. Sections were prepared and stained with H&E staining to observe whether there were lesions in important organs.

### Biodistribution analyses

In order to evaluate the biodistribution of FZ/MER-AgMOF@Bm in vivo, mice with established CLP model were randomly grouped, and injected with Cy5-labeled FZ/MER-AgMOF and Cy5-labeled FZ/MER-AgMOF@Bm via tail vein. The nanoparticles described above were injected into caudal vein at the dose of 1 µg/kg. Then, the fluorescence signals at 6, 12, 24 and 48 h after administration were detected by using the Xenogen IVIS Lumina XR imaging system (Caliper Life Sciences, USA).

### In vivo efficacy of FZ/MER-AgMOF@Bm

Mice were randomly allocated to the control (sham operation), CLP, CLP + AgMOF, CLP + AgMOF@Bm, CLP + FPS-ZM1, CLP + meropenem, CLP + FZ/MER-AgMOF and CLP + FZ/MER-AgMOF@Bm groups. There were 14 mice in the control group (4 for survival analysis) and 18 mice in each treatment group (8 for survival analysis). CLP challenge 2 h later, mice were treated with 0.9% saline (i.v., CLP group), AgMOF (i.v.,7.5 mg/kg), AgMOF@Bm (7.5 mg/kg), FPS-ZM1 (i.v., 1.5 mg/kg), meropenem (i.v., 1.5 mg/kg), FZ/MER-AgMOF (i.v., FPS-ZM1 dose of 1.5 mg/kg) or FZ/MER-AgMOF@Bm (i.v., FPS-ZM1 dose of 1.5 mg/kg). The body temperature of mice was captured at different points in time (0, 2, 6 and 12 h) after drug treatment. For the survival test, mice were monitored 3 times daily for a total of 6 days. 24 h after drug treatment, the serum levels of IL-1β, IL-6 and IL-10 were measured by using ELISA kits. The major organs were collected for H&E staining and immunohistochemistry (IHC) for histopathological analysis. Besides, 300 µL of serum was used for spread plate to detect bacterial content in mouse blood. Mouse peritoneal lavage fluid was obtained, and OD_600_ values of the lavage fluid were measured using a UV spectrophotometer to estimate bacterial density.

### Statistical analysis

Statistical analysis was performed through GraphPad Prism software, and data expressed as mean ± SD. Differences between groups were assessed by one way ANOVA with subsequent Tukey’s post-test (*P < 0.05, **P < 0.01, ***P < 0.001). Kaplan-Meier survival plots were plotted to compare survival differences between treatment groups.

## Electronic supplementary material

Below is the link to the electronic supplementary material.

Supplementary Material 1

Supplementary Material 2

## Data Availability

All data generated or analyzed during this study are included in this published article.
