# Supplementary material for: Bioengineered stem cell membrane functionalized nanoparticles combine anti-inflammatory and antimicrobial properties for sepsis treatment
Source: J Nanobiotechnology. 2023 May 26;21:170. doi: 10.1186/s12951-023-01913-3 (PMC10214628; doi:10.1186/s12951-023-01913-3)
Supplement: Supplementary file 1 — Supplementary Material 1 [file 12951_2023_1913_MOESM1_ESM.pdf]

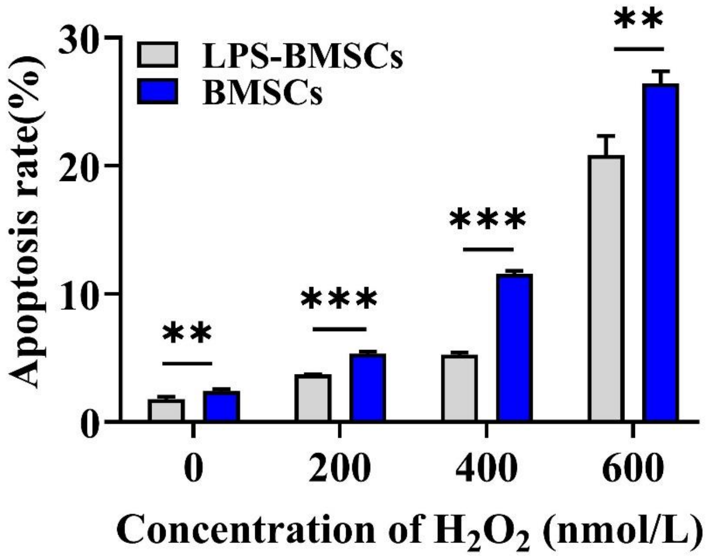

Fig. S1 Flow cytometry was used to quantitatively analyze the apoptosis rate of BMSCs and LPS-BMSCs treated with H_2O_2 for 24 h. Data are derived from three independent experiments and presented as mean ± SEM in the bar graphs. $^{**}P < 0.01$, $^{***}P < 0.001$.

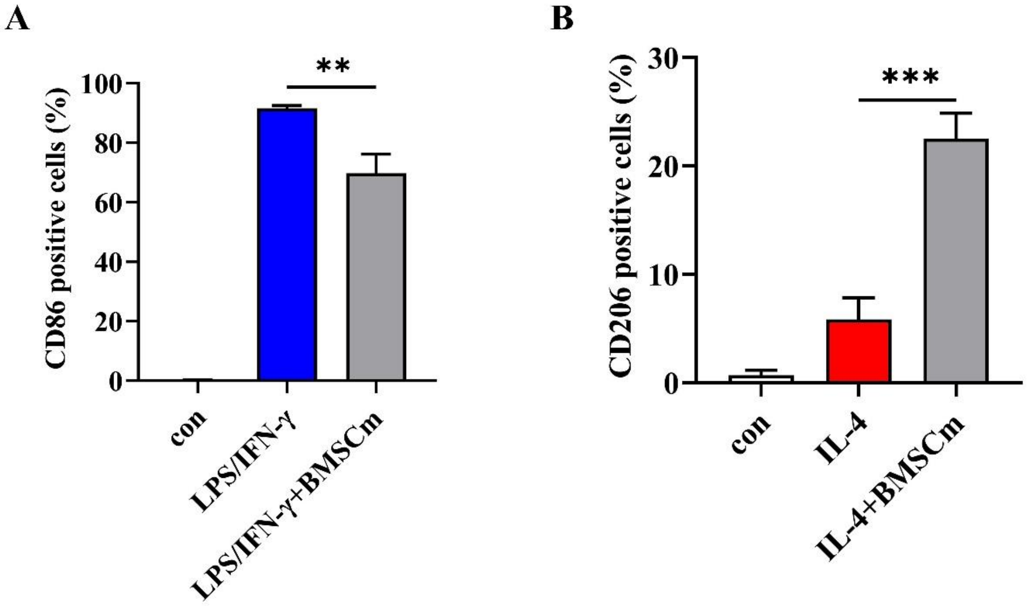

Fig. S2 Quantitative analysis by flow cytometry showed the fluorescence intensity of CD86 **(A)** and CD206 **(B)** in RAW264.7. RAW264.7 stimulated with LPS (100 ng/mL)

+ IFN-γ (20 ng/mL) or IL-4 (20 ng/mL) treated with or without LPS-BMSC membrane vesicles (BMSCm) for 24 h. Data are derived from three independent experiments and presented as mean ± SEM in the bar graphs. $^{**}P < 0.01$, $^{***}P < 0.001$.

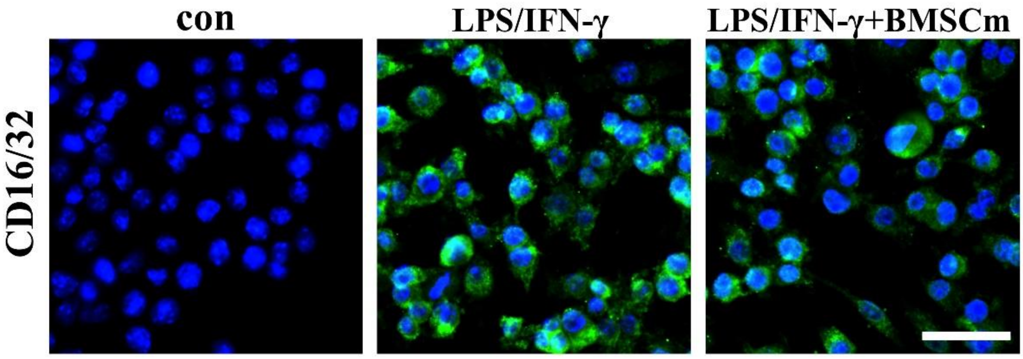

Fig. S3 M1 macrophage markers CD16/32 were examined by immunofluorescence. RAW264.7 stimulated with LPS (100 ng/mL) + IFN-γ (20 ng/mL) treated with or without LPS-BMSC membrane vesicles (BMSCm) for 24 h. Blue: DAPI; Green: CD16/32; Scale bar: 50 μm.

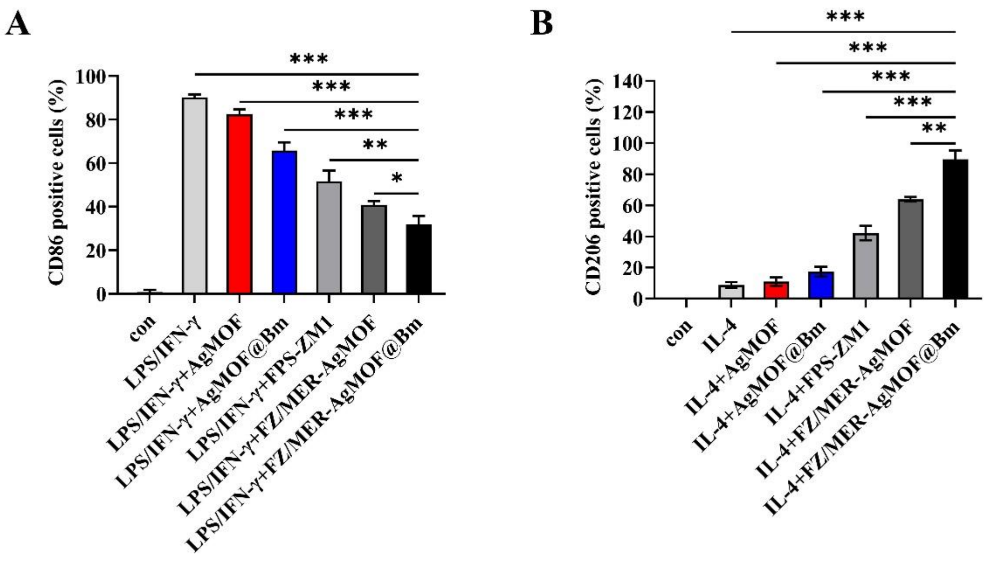

Fig. S4 Quantitative analysis by flow cytometry showed the fluorescence intensity of

CD86 **(A)** and CD206 **(B)** in RAW264.7. RAW264.7 stimulated with LPS (100 ng/mL) + IFN-γ (20 ng/mL) or IL-4 (20 ng/mL) treated indicated reagents for 24 h. Data are derived from three independent experiments and presented as mean ± SEM in the bar graphs. **P* < 0.05, ***P* < 0.01, ****P* < 0.001.

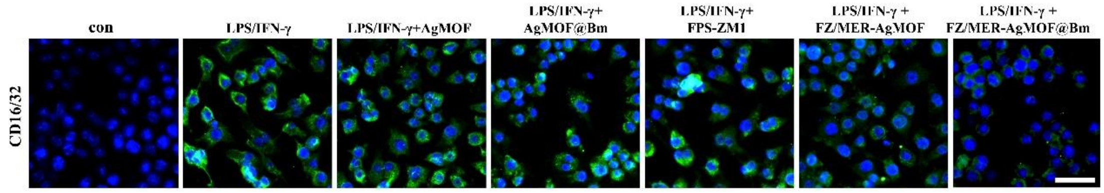

Fig. S5 M1 macrophage markers CD16/32 were examined by immunofluorescence. RAW264.7 were stimulated with LPS (100 ng/mL) + IFN-γ (20 ng/mL) for M1-like polarization and treated indicated reagents for 24 h. Blue: DAPI; Green:CD16/32; Scale bar: 50 μm.

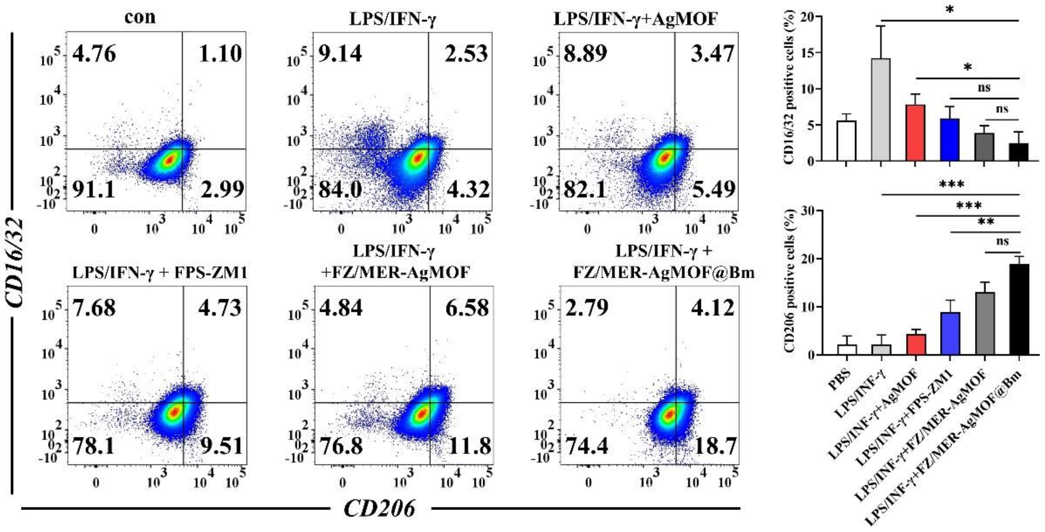

Fig. S6 Flow cytometry evaluated macrophage subsets by staining CD16/32 and CD206. RAW264.7 were stimulated with LPS (100 ng/mL) + IFN-γ (20 ng/mL) for 24

h for M1-like polarization and then treated with indicated reagents for additional 24 h. Data are derived from three independent experiments and presented as mean ± SEM in the bar graphs. *$P < 0.05$, **$P < 0.01$, ***$P < 0.001$, ns: not significant.

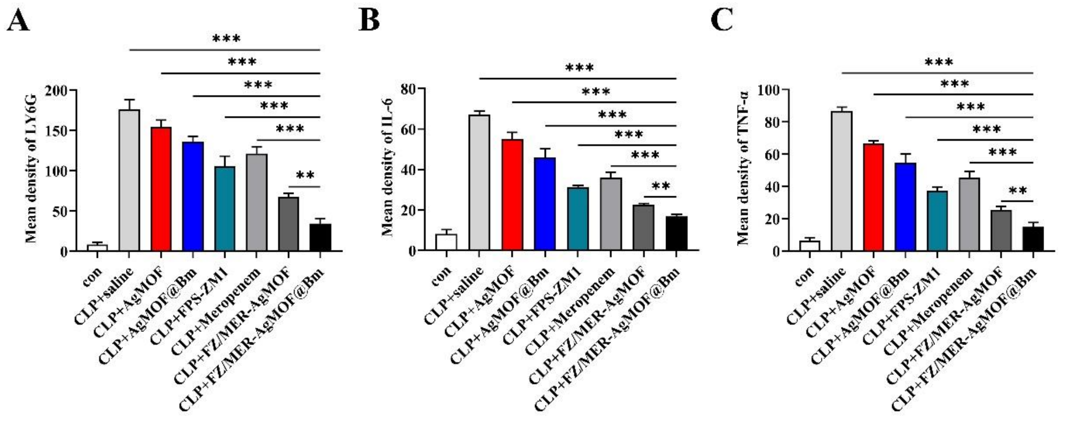

Fig. S7 Intensity quantification for IHC showing the expression levels of LY6G **(A)**, TNF-α **(B)** and IL-6 **(C)** in lungs. Data are derived from three independent experiments and presented as mean ± SEM in the bar graphs. **$P < 0.05$, ***$P < 0.001$.
