# Supplementary figures and images for: Bioengineered stem cell membrane functionalized nanoparticles combine anti-inflammatory and antimicrobial properties for sepsis treatment
Source: J Nanobiotechnology. 2023 May 26;21:170. doi: 10.1186/s12951-023-01913-3 (PMC10214628; doi:10.1186/s12951-023-01913-3)

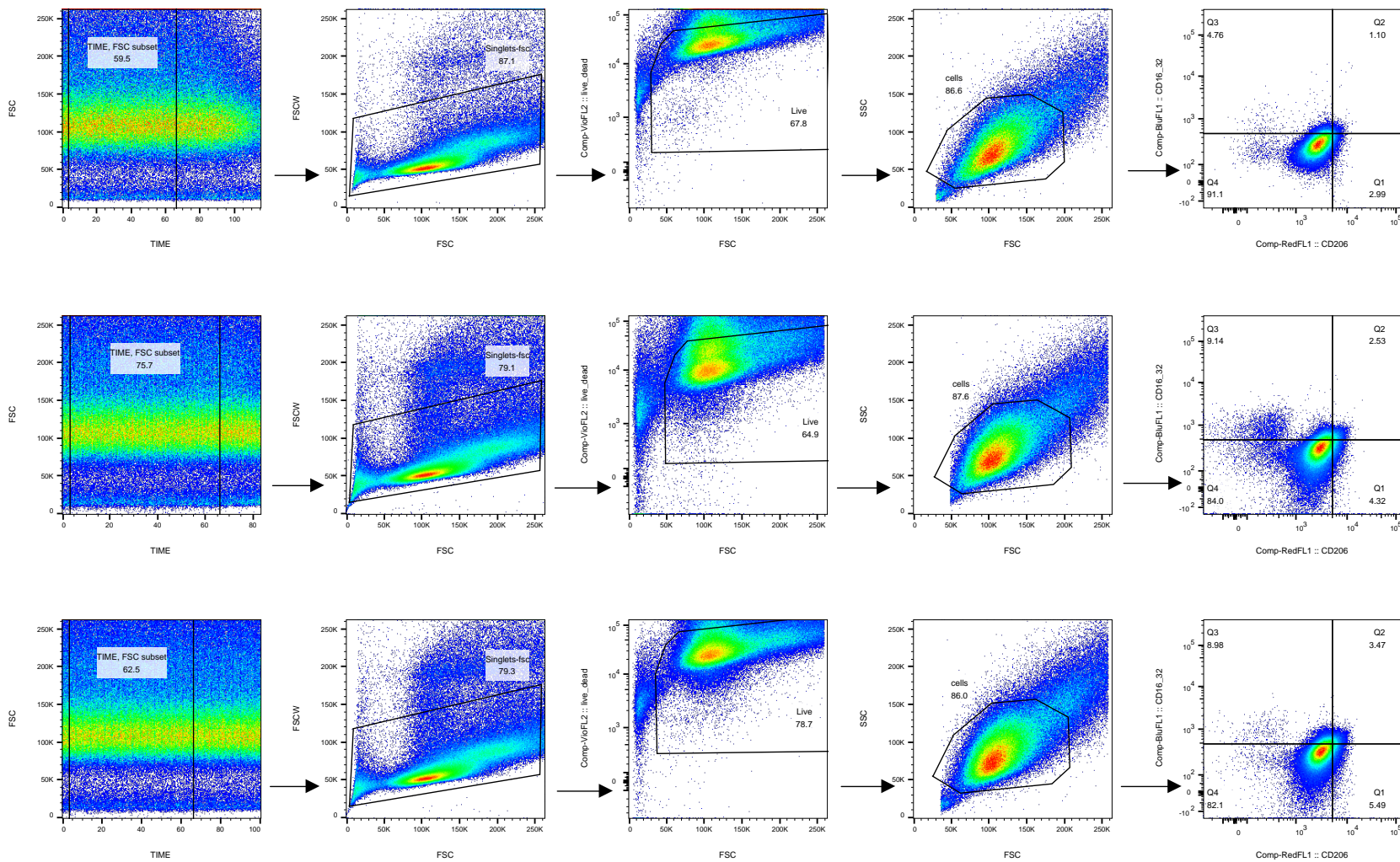

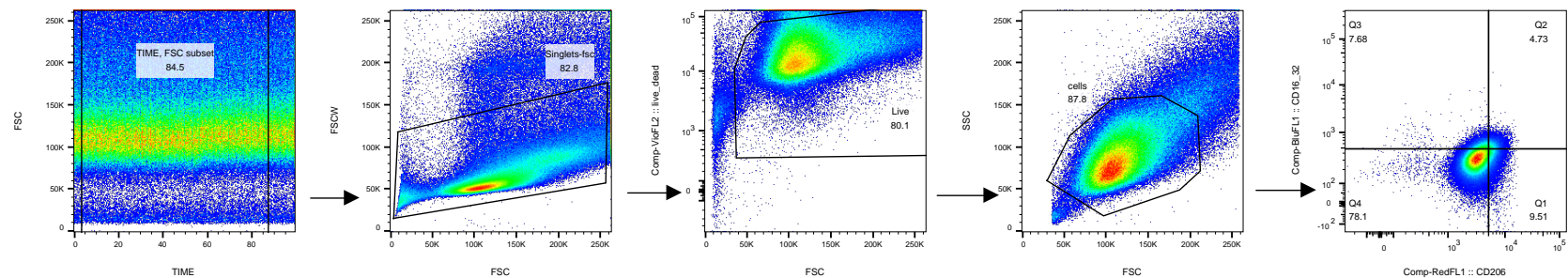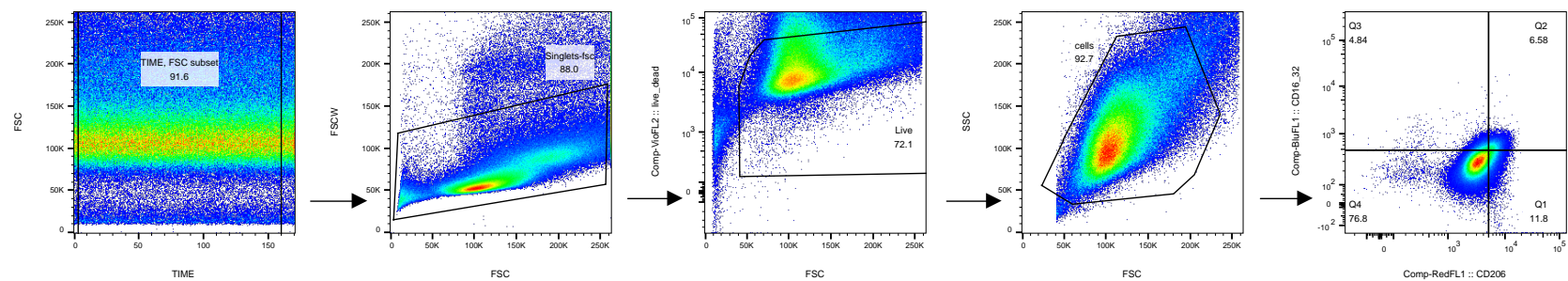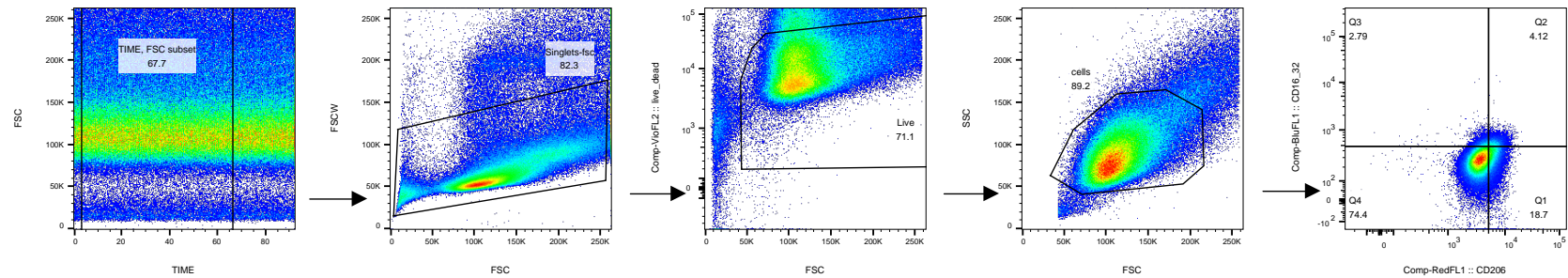

Supplement: Supplementary file 2 — Supplementary Material 2 [file 12951_2023_1913_MOESM2_ESM.pdf]
